# Shear stress associated with cardiopulmonary bypass induces expression of inflammatory cytokines and necroptosis in monocytes

**DOI:** 10.1172/jci.insight.141341

**Published:** 2021-01-11

**Authors:** Lan N. Tu, Lance Hsieh, Masaki Kajimoto, Kevin Charette, Nataliya Kibiryeva, Adriana Forero, Sarah Hampson, Jennifer A. Marshall, James O’Brien, Marta Scatena, Michael A. Portman, Ram Savan, Chris Benner, Alberto Aliseda, Muhammad Nuri, Douglas Bittel, Peter Pastuszko, Vishal Nigam

**Affiliations:** 1 Department of Pediatrics, Cardiology, University of Washington, Seattle, Washington, USA.; 2 Center for Developmental Biology and Regenerative Medicine, and; 3 Center for Integrative Brain Research, Seattle Children’s Research Institute, Seattle, Washington, USA.; 4 Department of Surgery, Pediatric Cardiac Surgery, University of Washington, Seattle, Washington, USA.; 5 Ward Family Heart Center, Children’s Mercy Hospital, Kansas City, Missouri, USA.; 6 Department of Immunology and; 7 Department of Bioengineering, University of Washington, Seattle, Washington, USA.; 8 Department of Medicine, UCSD, La Jolla, California, USA.; 9 Department of Mechanical Engineering, University of Washington, Seattle, Washington, USA.; 10 College of Biosciences, Kansas City University of Medicine and Biosciences, Kansas City, Missouri, USA.; 11 Department of Cardiovascular Surgery, Icahn School of Medicine at Mount Sinai, New York, New York, USA.

**Keywords:** Cardiology, Inflammation, Calcium signaling, Cytokines, Surgery

## Abstract

Cardiopulmonary bypass (CPB) is required during most cardiac surgeries. CBP drives systemic inflammation and multiorgan dysfunction that is especially severe in neonatal patients. Limited understanding of molecular mechanisms underlying CPB-associated inflammation presents a significant barrier to improve clinical outcomes. To better understand these clinical issues, we performed mRNA sequencing on total circulating leukocytes from neonatal patients undergoing CPB. Our data identify myeloid cells, particularly monocytes, as the major cell type driving transcriptional responses to CPB. Furthermore, IL-8 and TNF-α were inflammatory cytokines robustly upregulated in leukocytes from both patients and piglets exposed to CPB. To delineate the molecular mechanism, we exposed THP-1 human monocytic cells to CPB-like conditions, including artificial surfaces, high shear stress, and cooling/rewarming. Shear stress was found to drive cytokine upregulation via calcium-dependent signaling pathways. We also observed that a subpopulation of THP-1 cells died via TNF-α–mediated necroptosis, which we hypothesize contributes to post-CPB inflammation. Our study identifies a shear stress–modulated molecular mechanism that drives systemic inflammation in pediatric CPB patients. These are also the first data to our knowledge to demonstrate that shear stress causes necroptosis. Finally, we observe that calcium and TNF-α signaling are potentially novel targets to ameliorate post-CPB inflammation.

## Graphical abstract

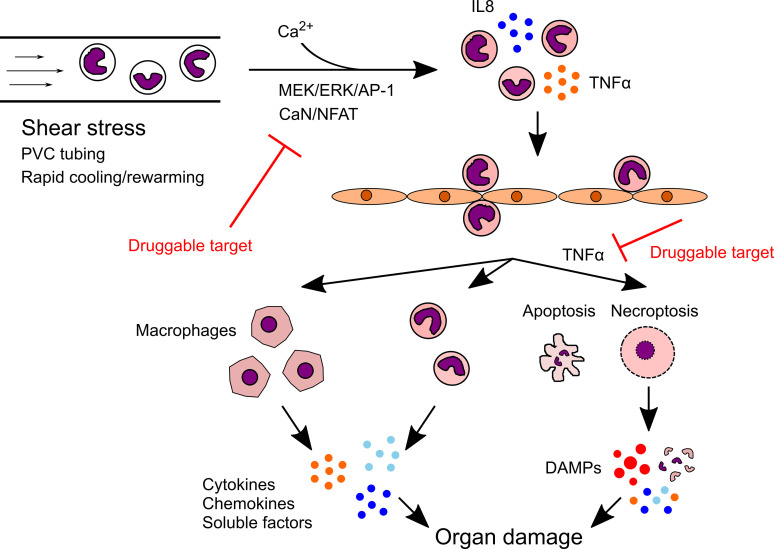

## Introduction

Congenital heart disease is the most common birth defect, with 0.6%–1.9% of live births having moderate or severe defects (1). Within the first year of life, approximately 25% of the affected infants require open-heart surgeries (2), which are associated with high mortality and complication rates of 10.7% and 29.2%, respectively (3). During the majority of cardiac surgeries, the patient is placed on cardiopulmonary bypass (CPB) in order to minimize ischemic damage while the heart is being operated on. CPB involves pumping the patient’s blood through an artificial “heart-lung” machine that exposes the blood to high shear stress and plastic tubing; the blood and patient are cooled during the surgery to minimize metabolic demands and then quickly rewarmed at the end of surgery. Exposure to CPB results in systemic inflammation and multiorgan dysfunction that is especially severe in neonatal and pediatric patients. Changes to clinical practice such as administration of corticosteroids (4), coating the CPB tubing (5), and performing modified ultrafiltration (MUF) (6), have failed to significantly reduce post-CPB complications.

Adverse postoperative outcomes are linked to the profound systemic inflammatory response during and after CPB surgery. A surge of proinflammatory cytokines — specifically IL-1β, IL-6, IL-8, and TNF-α — have consistently been seen in the plasma of CPB patients (7, 8). Systemic inflammation has been tied to longer intensive care unit and hospital stays (9), as well as postoperative complications such as organ damage and dysfunction (10). Specifically, elevated IL-8 levels in neonatal patients were associated with prolonged hospital stay (9), myocardial injury (8, 11), and length of mechanical ventilation (12). High levels of TNF-α after CPB are associated with reduced glomerular filtration rate and renal insufficiency (13). Given how severe the systemic inflammatory response is and the important role it plays in clinical outcomes of pediatric patients, it is critical to elucidate the molecular mechanisms by which CPB induces inflammatory cytokine release. This is the first key step to identify novel targets for therapeutic interventions.

Although the link between systemic inflammation and organ damage has been strongly implicated, the mechanism of how this occurs remains unclear. One leading hypothesis is that CPB activates inflammatory leukocytes that then extravasate and infiltrate into different organs after CPB, causing organ dysfunction. CPB has been shown to induce leukocyte adhesion and transmigration in patients after surgery (14); the accumulation of leukocytes in various organs after surgery has also been confirmed in CPB animal models (15, 16). The question then becomes how infiltrated leukocytes cause damage. Inflammatory leukocytes, including differentiated macrophages, release cytokines and damaging soluble factors to the surrounding tissues (16). These cells might also die upon infiltration, as CPB and particularly high shear stress were shown capable of inducing cell death in monocytes (17, 18). Necrosis, the inflammatory mode of cell death, is considered more damaging than apoptosis, as cells rupture and release all intracellular contents called damage-associated molecular patterns (DAMPs) into the environment. Regulated necrosis has been shown to critically regulate many disease pathologies, with receptor-interacting protein kinase–mediated (RIPK-mediated) necroptosis (19) and inflammasome-mediated pyroptosis (20) being the most well characterized. To date, the experimental evidence for the death of CPB-activated leukocytes and the exact mode of cell death, if it indeed occurs, is still lacking.

Our study used pediatric patient samples, a piglet CPB model, and a newly developed in vitro CPB model to characterize the molecular mechanisms of how CPB and its specific factors induce systemic inflammation. We identified high shear stress as a CPB-specific factor sufficient to markedly upregulate IL-8 and TNF-α production in human monocytes via calcium-dependent signaling pathways. A significant subpopulation of CPB-induced inflammatory monocytic cells died by both apoptosis and TNF-α–mediated necroptosis, which could contribute to the post-CPB tissue injuries.

## Results

### CPB activates myeloid cells to increase the production of IL-8 and TNF-α.

**Figure 1:**
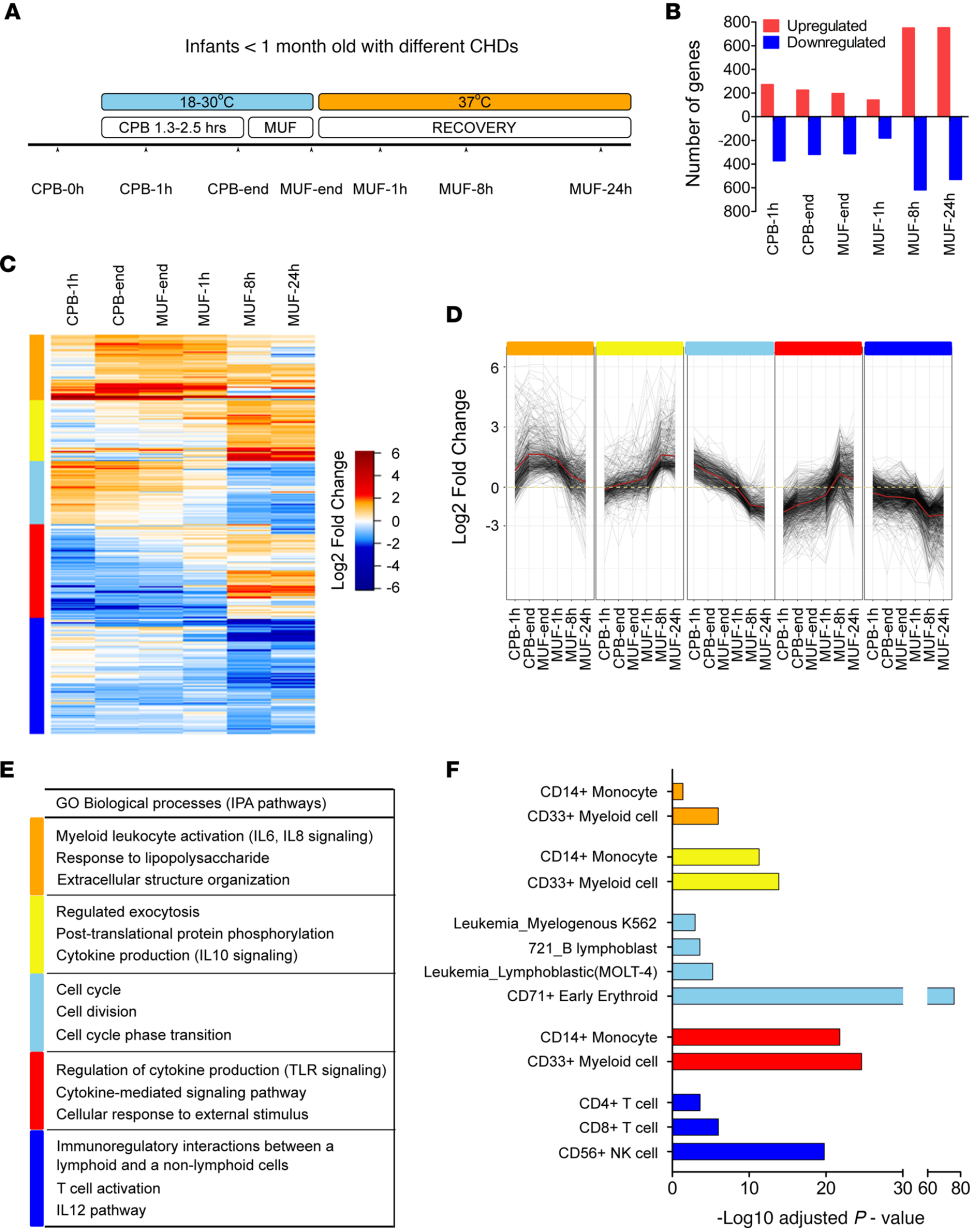
mRNA-Seq analysis of total nucleated cells from pediatric patients undergoing CPB. **(A**) The design and time course of the study. Infants with congenital heart diseases (CHDs) underwent CPB surgery that lasted 1.3–2.5 hours on average, followed by a short duration of modified ultrafiltration (MUF). Body temperature was cooled to 18°C–30°C during surgery and quickly rewarmed to 37°C after MUF. Blood samples were collected at 7 time points for mRNA-Seq of total nucleated cells (*n* = 5 patients). (**B**) The number of genes that were significantly downregulated or upregulated at different time points compared with the expression level before surgery (CPB-0h) for each patient. Significance was defined as at least 2-fold change with adjusted *P* < 0.01. (**C**) Identification of 5 clusters by hierarchical clustering of 2686 differentially expressed genes that were significant at all time points based on Euclidean distances. (**D**) Kinetics of gene expression within distinct gene expression clusters. Dotted line indicates mean gene expression in each cluster. (**E**) Pathway enrichment analysis by Metascape and Ingenuity Pathway Analysis (IPA) identified top gene ontology (GO) biological processes being enriched in each cluster. (**F**) Computational deconvolution of cell subsets in each cluster using GeneAtlas in EnrichR.

Neonatal cardiac surgery patients were enrolled in our study (patient demographics in Supplemental Table 1; supplemental material available online with this article; https://doi.org/10.1172/jci.insight.141341DS1). Nucleated cells, predominantly leukocytes, were isolated from whole blood collected at 7 time points before, during, and after surgery (Figure 1A). Total mRNA was used for mRNA sequencing (mRNA-Seq), and the baseline level of gene expression before CPB (CPB-0h) was used to calculate fold changes in gene expression at other time points for each patient. We defined significant differentially expressed (DE) genes as those with at least 2-fold change in expression with an adjusted *P* < 0.01. The total number of DE genes at each time point was as follows: CPB at 1 hour (CPB-1h): 271 genes upregulated (up), 372 genes downregulated (down); CPB-end: 224 up, 319 down; MUF-end: 196 up, 312 down; MUF-1h: 141 up, 180 down; MUF-8h: 749 up, 618 down; MUF-24h: 751 up, 530 down (Figure 1B). A total of 2686 DE genes was significant across all the time points and was included in further analysis. Hierarchical clustering of these genes based on relative gene expression identified 5 distinct clusters: orange cluster for genes that strongly upregulated at all time points, yellow cluster for genes that upregulated progressively over time, light blue cluster for genes whose expressions went up and then down, red cluster for genes whose expressions went down and then up, and blue cluster for genes that downregulated at all time points (Figure 1, C and D). Pathway enrichment analysis for genes in each cluster showed that there was strong activation of myeloid cells (orange cluster) at all time points, particularly for granulocyte adhesion and IL-6 and IL-8 signaling (Figure 1E and Supplemental Tables 3 and 4). Cell proliferation (light blue cluster) was prominent only during surgery (Figure 1E and Supplemental Table 3). There was a gradual increase over time in exocytosis, antiinflammatory cytokine signaling mediated by IL-10, TLR signaling, and leukocyte migration (yellow and red clusters) (Figure 1E and Supplemental Table 3). Signaling involving lymphocytes was strongly downregulated at all time points (blue cluster) (Figure 1E and Supplemental Table 3).

Cell type–specific gene set enrichment analysis was performed using the Gene Atlas database (http://amp.pharm.mssm.edu/Enrichr) (Figure 1F and Supplemental Figure 1A). Myeloid cells, especially monocytes, were found to be the main cell type driving the overall transcriptional response. CD71^+^ erythroid cells, immature nucleated RBC isolated with the leukocytes, were dominant in the light blue cluster and particularly at the CPB-1h time point, likely contributing to the cell proliferation phenotype. The involvement of lymphocytes and NK cells seemed to be minor. These results further corroborated the pathway enrichment analysis result above. In addition, analysis of upstream regulatory kinases predicted strong activation of many kinases in the MEK/ERK pathway (Supplemental Figure 1B).

**Figure 2:**
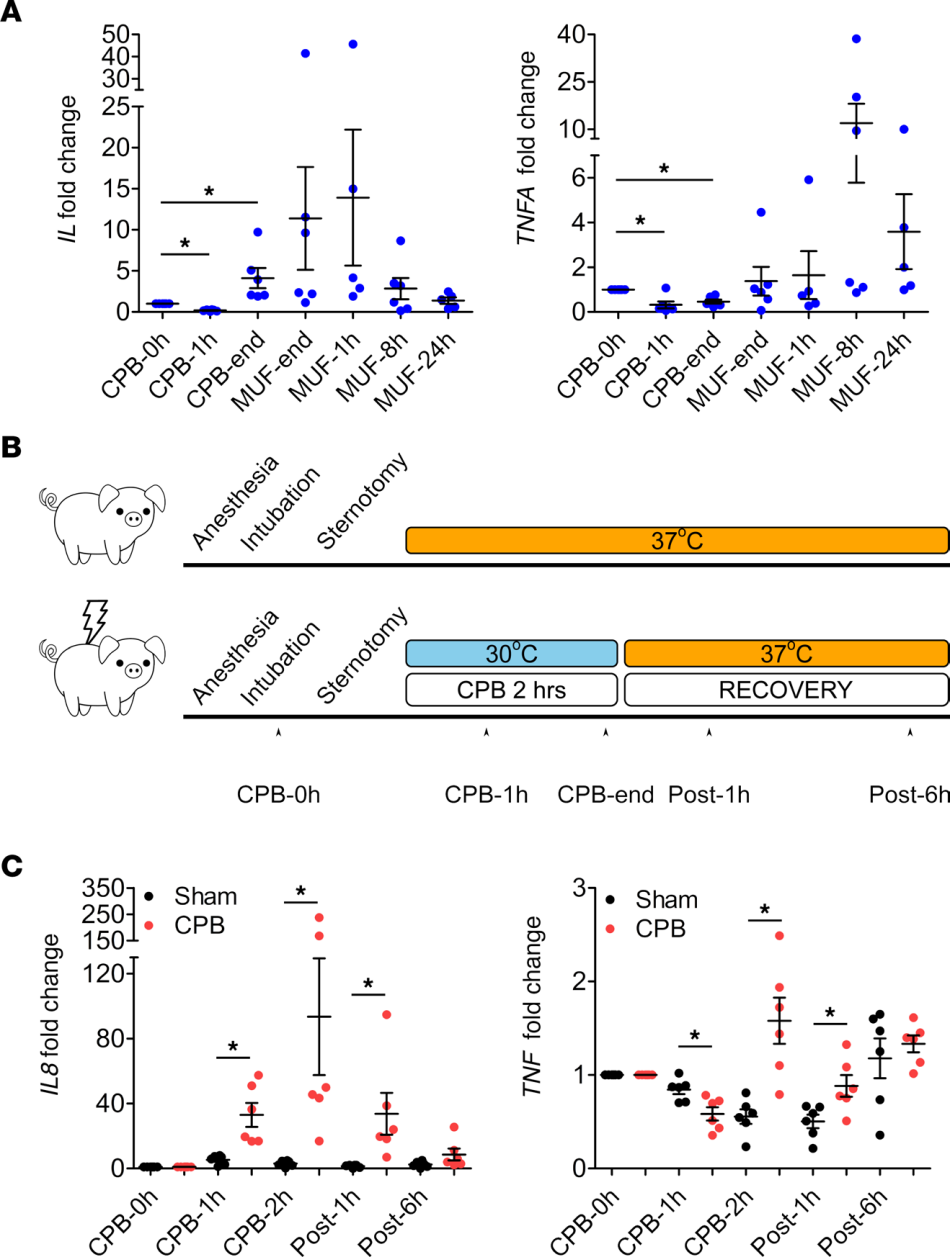
Leukocytes specifically upregulate *IL8* and *TNFA* in pediatric patients and piglet CPB model. (**A**) qPCR analysis from total leukocytes of pediatric patients undergoing CPB showed that the level of *IL8* and *TNFA* trended higher at the end of CPB and during recovery, but the values were not significant (*n* = 5–6 patients, an independent cohort). Kruskal-Wallis test. (**B**) The design and time course of the piglet CPB model. Healthy 1-month-old male piglets underwent CPB surgery for 2 hours; body temperature was cooled to 30°C during surgery and quickly rewarmed to 37°C for recovery. Sham piglets had body temperature maintained at 37°C throughout the study. Blood samples were collected at 5 time points (*n* = 6 piglets/group). (**C**) qPCR analysis showed significant upregulation of *IL8* and *TNF* in the leukocytes from CPB piglets compared with the sham piglets (*n* = 6 piglets/group). *P < 0.05, Kruskal-Wallis and post hoc Dunn’s test for *IL8*; 1-way ANOVA and post hoc Tukey’s test for *TNF*.

Since inflammatory cytokines are the hallmark of the systemic inflammation in pediatric patients after CPB, we analyzed the transcriptional changes of 4 cytokines: *IL1B*, *IL6*, *IL8*, and *TNFA* in the mRNA-Seq data and only found upregulation of *IL8* and *TNFA*; no *IL6* transcript was detected (Supplemental Figure 1C). Quantitative PCR (qPCR) was then performed using mRNA of leukocytes from an independent cohort of 6 pediatric patients distinct from those used for mRNA-Seq (patient demographics in Supplemental Table 1). *IL8* and *TNFA* mRNA levels trended higher at the end of CPB and after surgery compared with pre-CPB; however, the values were not statistically significant due to high variations in the fold changes among patients (Figure 2A). *IL1B* expression was not altered (Supplemental Figure 2A), while *IL6* was not detected (not shown). Since such inflammatory response could be CPB specific or due to factors such as blood transfusion, drugs, or a patient’s specific conditions, we sought to determine if CPB alone upregulates *IL8* and *TNFA* production. We utilized a piglet CPB model using healthy 1-month-old piglets (*n* = 6 piglets/group). Both sham and bypass piglets were anesthetized, intubated, underwent median sternotomy, and received similar drugs and fluids throughout the procedure. Bypass piglets were not exposed to blood transfusion. Leukocytes were isolated from the piglets at 5 time points, before, during, and after surgery (Figure 2B). qPCR confirmed that both *Il8* and *Tnf* were significantly upregulated in the bypass piglets compared with the sham controls (Figure 2C). *Il6* and *Il1b* mRNA levels were not different between the 2 groups (Supplemental Figure 2, B and C). We conclude that exposure to CPB conditions is sufficient to upregulate *IL8* and *TNFA* expression in leukocytes.

### Contribution of different CPB-specific factors in upregulating IL-8 and TNF-α in monocytes.

**Figure 3:**
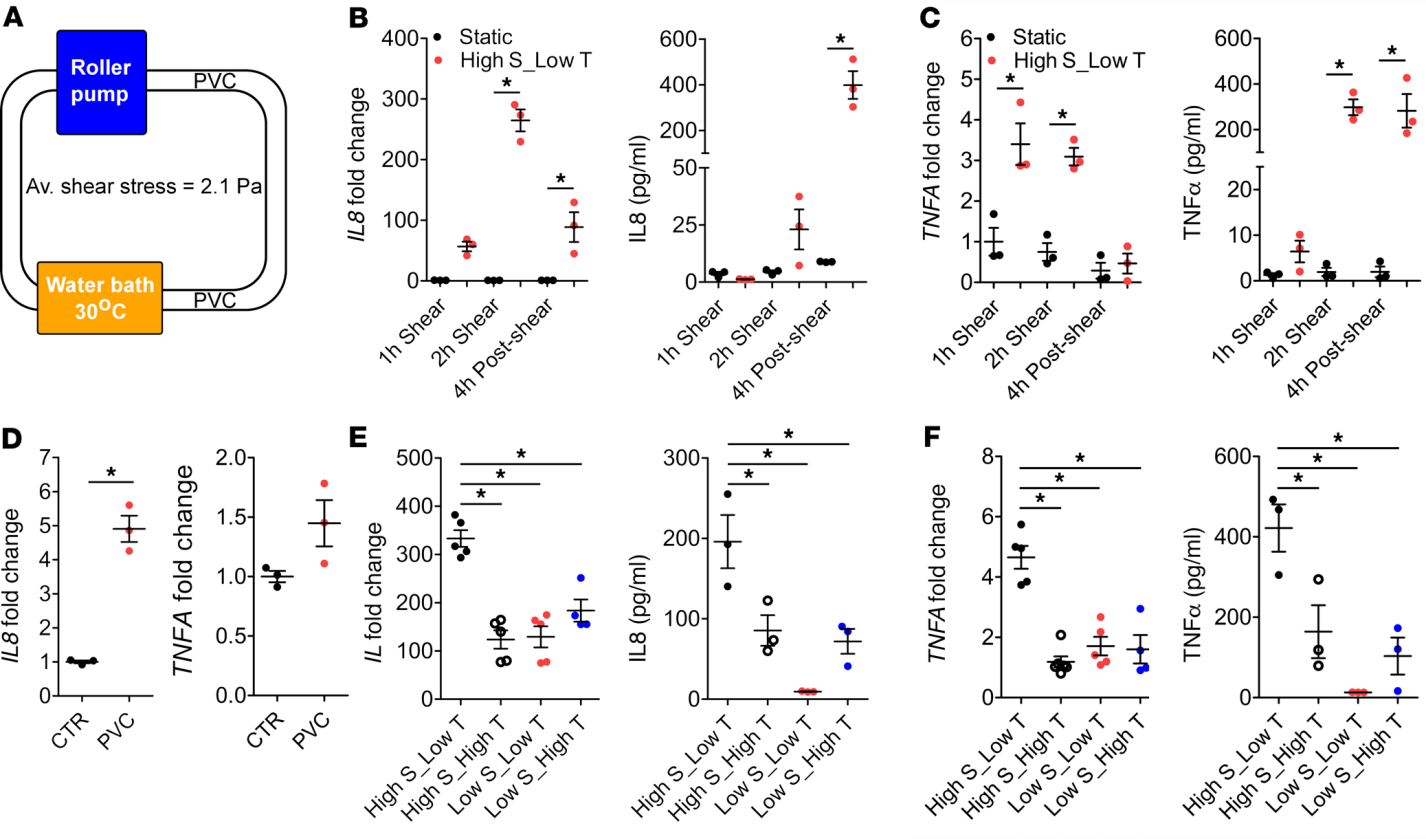
In vitro CPB conditions upregulate IL-8 and TNF-α in THP-1 cells. (**A**) The design of in vitro CPB system. THP-1 cells at a density of 2 million cells/mL were pumped by a roller pump through PVC tubing at an average shear stress of 2.1 Pa. The majority of the tubing was kept in the water bath that maintained the temperature at 30°C. Cells were warmed up to 37°C for recovery. (**B** and **C**) The CPB conditions of high shear stress, low temperature (High S_Low T) significantly upregulated the mRNA and protein levels of IL-8 (**B**) and TNF-α (**C**) in the sheared THP-1 cells compared with static cells (*n* = 3 replicates/group). *P < 0.05, 1-way ANOVA and post hoc Tukey’s test. (**D**) Static THP-1 cells incubated with PVC tubing for 2 hours significantly upregulated *IL8* by approximately 5-fold. Level of *TNFA* was not changed (*n* = 3 replicates/group). *P < 0.05, 2-tailed Student’s *t* test. (**E** and **F**) When THP-1 cells were sheared at 37°C (High S_High T) or at low shear stress of 0.67 Pa (Low S_Low T and Low S_High T), upregulation of mRNA and protein levels of IL-8 (**E**) and TNF-α (**F**) were significantly reduced compared with when cells were sheared at CPB conditions (High S_Low T) (*n* = 3–5 replicates/group). *P < 0.05, 1-way ANOVA and post hoc Dunnett’s test.

To investigate the molecular mechanisms by which CPB induces cytokine production in monocytes, we exposed THP-1 cells, a human monocytic cell line from an infant, to in vitro CPB conditions that include a roller pump, polyvinyl chloride (PVC) tubing, and cooling/rewarming (Figure 3A). Cells were exposed to shear conditions for a duration of 2 hours, unless otherwise indicated, at the temperature of 30°C; they were then warmed to 37°C. In this in vitro CPB system, THP-1 cells were subjected to a wide range of shear stress values; the sheared cells experienced shear varying across the cross-sectional area of the tubing, from 0 at the centerline to a maximum of 4.2 Pa at the wall, with an average shear stress of 2.1 Pa (twice the average physiological value in the systemic circulation). While no cells experience the maximum value of shear at the wall, most cells flow in the area that is near the wall (75% of the cells will be, at any given instant, in a region where they are exposed to a shear between the mean of 2.1 Pa and the maximum of 4.2 Pa). This level of shear stress was chosen to reproduce the mean exposure of leukocytes in the CBP system, with a perfusion rate of 150 mL/kg/min, for a 6.2 kg patient, and a 6-Fr arterial cannula, 8-Fr venous cannula, and 3/16” and ¼” arterial and venous lines, respectively. The design of the in vitro system, therefore, recapitulates conditions present in CPB circuits used in pediatric patients and the piglet CPB model (Supplemental Figure 2D). First, we confirmed that the in vitro CPB conditions robustly upregulated both transcript and protein levels of IL-8 and TNF-α in THP-1 cells compared with the static control (Figure 3, B and C). *IL1B* expression was not significantly altered (Supplemental Figure 3A), and no changes in IL-1B and IL-6 protein levels were detected in the sheared THP-1 cells (not shown). In addition, when human primary CD14^+^ monocytes were subjected to the same in vitro CPB conditions, IL-8 and TNF-α were significantly elevated compared with the static cells (Supplemental Figure 3B). These data support our conclusion that the in vitro CPB circuit recapitulates the in vivo CPB data in that it upregulates IL-8 and TNF-α.

Three factors associated with the CPB circuit are exposure to PVC, high shear stress, and rapid cooling/rewarming. To dissect out the effect of each component, we incubated static THP-1 cells with PVC tubing for 2 hours and found that exposure to PVC alone at 37°C upregulated *IL8* by approximately 5-fold (Figure 3D), while the level of *TNFA* was not significantly altered (Figure 3D). When the cells were sheared at 37°C instead of 30°C to eliminate the cooling/rewarming effect, or sheared at a lower shear stress of 0.63 Pa, the upregulation of IL-8 and TNF-α at both mRNA and protein levels was significantly reduced (Figure 3, E and F). Specifically for *IL8*, the CPB condition of high shear stress at 2.1 Pa and low temperature at 30°C increased *IL8* level by approximately 330-fold compared with the static control (Figure 3E). When cells were exposed to high shear stress at 2.1 Pa at 37°C, the *IL8* was upregulated approximately 124-fold compared with the static control (Figure 3E), suggesting that the effect of cooling/rewarming accounted for approximately 2.7-fold difference in the *IL8* level. With PVC exposure upregulating *IL8* by only approximately 5-fold, we speculate that shear stress contributes the most to the 330-fold increase in *IL8* mRNA levels in the sheared THP-1 cells compared with the static condition. This comparison demonstrates the dominant effect of shear stress among the 3 CPB-specific factors but does not take into account the possibility of synergism. Besides, cell density showed no effect on the *IL8* level, while the *TNFA* level was significantly more elevated when cells were sheared at densities higher than 1 million cells/mL (Supplemental Figure 3C).

### CPB activates calcium-dependent signaling pathways in monocytes.

**Figure 4:**
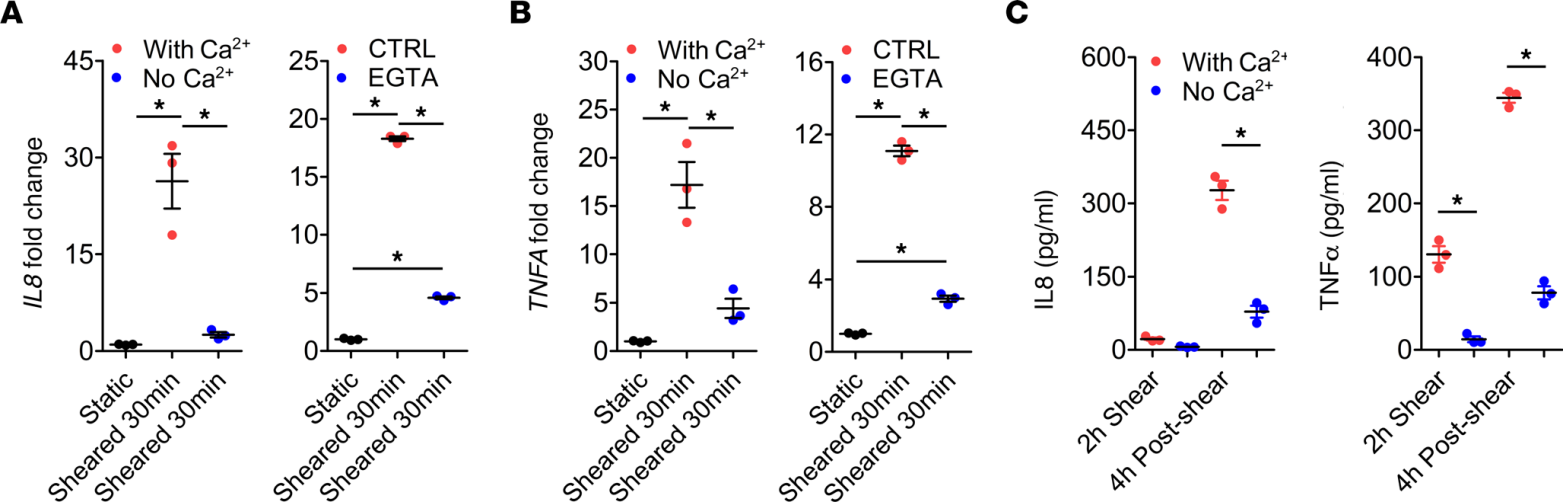
Extracellular calcium is essential for CPB-induced upregulation of IL-8 and TNF-α. (**A** and **B**) When THP-1 cells were sheared in the calcium-free media or in the presence of EGTA, a calcium chelator, the mRNA upregulation of *IL8* (**A**) and *TNFA* (**B**) was markedly blunted compared with the respective control media (*n* = 3 replicates/group). *P < 0.05, 1-way ANOVA and post hoc Tukey’s test. (**C**) Protein levels of IL-8 and TNF-α in the THP-1 cells sheared in calcium-free media were significantly lower than those in the control media (*n* = 3 replicates/group). *P < 0.05, 1-way ANOVA and post hoc Tukey’s test.

**Figure 5:**
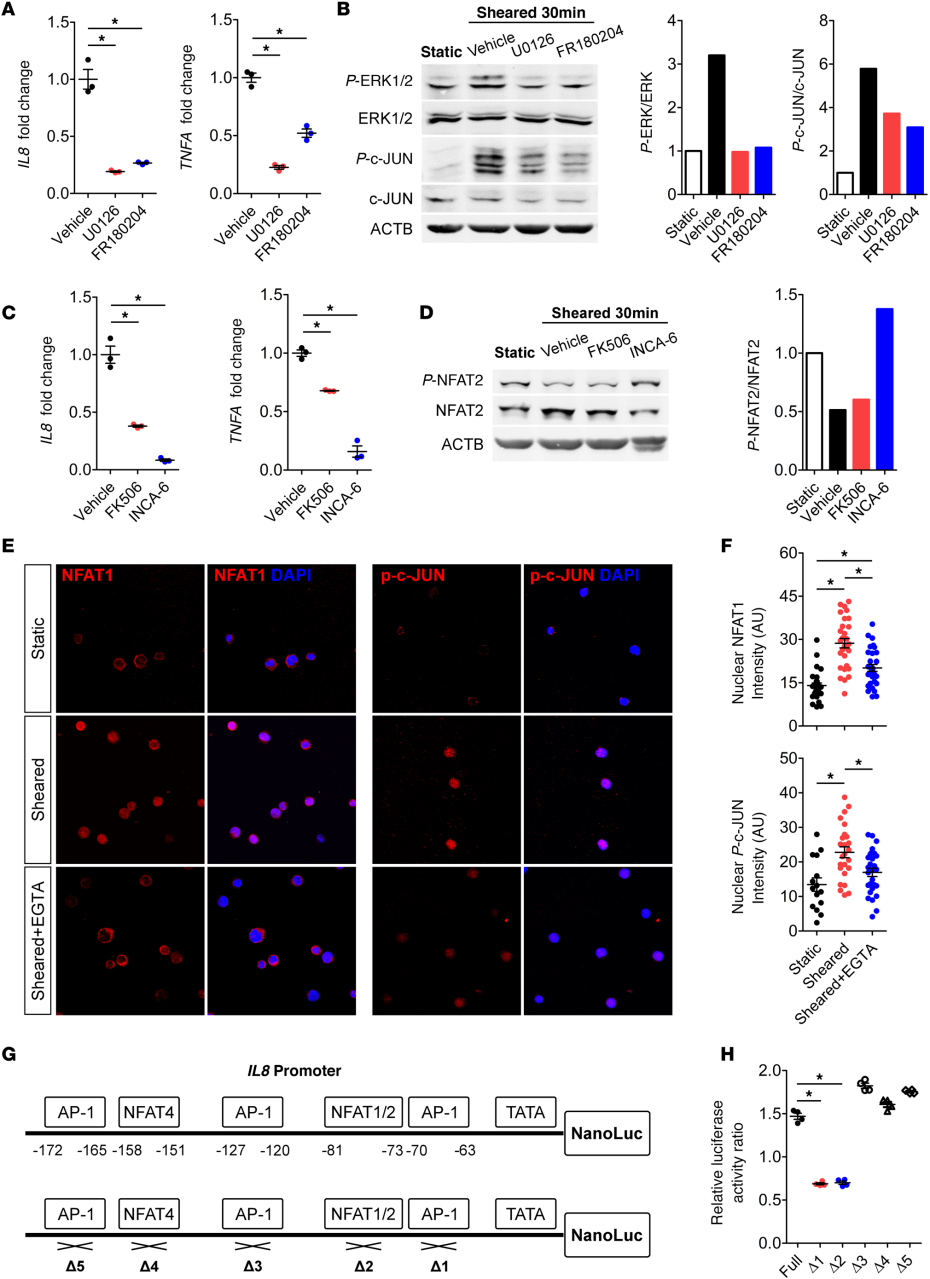
CPB conditions upregulate *IL8* and *TNFA* via MEK/ERK/AP-1 and CaN/NFAT pathways. (**A**) U0126 and FR180204 — inhibitors of MEK and ERK, respectively — significantly reduced upregulation of *IL8* and *TNFA* compared with the vehicle controls in THP-1 cells (*n* = 3 replicates/group). (**B**) Western blot showed increased levels of p-ERK1/2 and p–c-JUN in sheared THP-1 cells, which were inhibited by U0126 and FR180204. (**C**) FK506 and INCA-6, inhibitors of Calcineurin(CaN)/NFAT interaction, significantly reduced upregulation of *IL8* and *TNFA* compared with the vehicle controls (*n* = 3 replicates/group). (**D**) Western blot showed dephosphorylation of NFAT2 in sheared THP-1 cells, which was inhibited by INCA-6. (**E** and **F**) Immunocytochemical analysis of sheared THP-1 cells showed prominent expression and localization of NFAT1 and p–c-JUN in the cellular nuclei, which was inhibited by EGTA (*n* = 15–30 cells/group). These blots were run in parallel and contemporaneously on separate gels. (**G**) A total of 500 bp of the *IL8* promoter was cloned to drive expression of nanoluciferase. The schematic shows the positions of predicted AP-1 and NFAT1/2 binding sites in the *IL8* promoter and mutants with each deletion. (**H**) When THP-1 cells were sheared, activation of the full *IL8* promoter led to a 1.5-fold increase in the luciferase activity compared with the static cells. When the binding sites #1 or #2 were deleted, there was no increase in the luciferase activity in the sheared THP-1 cells (*n* = 4 replicates/group). *P < 0.05, 1-way ANOVA and post hoc Dunnett’s test (**A**, **C**, and **H**); 1-way ANOVA and post hoc Tukey’s test (**F**).

Since calcium signaling has been shown to play an important role in cellular responses to biomechanical stimuli, we investigated whether calcium is critical for CPB-activated cytokine production. When cells were sheared in the calcium-free media or when EGTA was used to chelate extracellular calcium, the CPB-induced upregulation of both mRNA and protein levels of IL-8 and TNF-α was markedly blunted compared with the respective control media (Figure 4), indicating that calcium influx into the cells is critical for such transcriptional response. We then investigated the specific calcium-mediated signaling pathways involved using chemical inhibitors specific to each pathway (Supplemental Figure 4A). THP-1 cells treated with U0126 or FR180204 to inhibit MEK and ERK, respectively, had drastically reduced upregulation of *IL8* and *TNFA* expression compared with the vehicle control (Figure 5A). Western blot result showed increased levels of phosphorylated ERK1/2 (p-ERK1/2) and p–c-JUN after shear, which were reversed by U0126 and FR180204 (Figure 5B). This result demonstrates activation of MEK/ERK pathway drives the expression of *IL8* and *TNFA* in response to CPB. Moreover, treatment of THP-1 cells with FK506 or INCA-6 to inhibit the interaction of Calcineurin (CaN) and NFAT also significantly reduced the upregulation of *IL8* and *TNFA* compared with the vehicle control (Figure 5C). Western blot result showed CPB-induced dephosphorylation of NFAT compared with the static condition, which was reversed by INCA-6 (Figure 5D). This indicates activation of the CaN/NFAT pathway by CPB. Western blot analysis of human primary CD14^+^ monocytes after shear also supported the activation of MEK/ERK/AP-1 and CaN/NFAT pathways (Supplemental Figure 3D). PKC inhibitors Go 6983 and Sotrastaurin could effectively inhibit the *IL8* and *TNFA* upregulation (Supplemental Figure 4B), but Western blot analysis did not show activation of the NF-κB pathway (Supplemental Figure 4C), suggesting that the inhibitory effect of PKC inhibitors was likely due to the crosstalk of PKC activating the MEK/ERK pathway. The CAMKII/CREB pathway is unlikely to be activated, since the CAMKII inhibitors STO-609 and CAMKIINtide did not consistently affect cytokine production and did not change the level of p-CAMKII (Supplemental Figure 4, D and E).

Since calcium signaling pathways can crosstalk and recruit different transcription factors (Supplemental Figure 4A), we examined nuclear localization of 4 transcription factors NFAT, AP-1 (p–c-JUN), NF-κB p65, and p-CREB in sheared THP-1 cells. Only NFAT1 and p–c-JUN showed clear nuclear localization in sheared THP-1 cells, which was inhibited by the calcium-chelator EGTA (Figure 5, E and F). No NF-κB p65 or p-CREB staining was detected in the nuclei of sheared THP-1 cells (Supplemental Figure 4, F and G). Therefore, we conclude that MEK/ERK/AP-1 and CaN/NFAT are the main calcium signaling pathways activated by CPB conditions. To further elucidate the binding sites of NFAT and AP-1 in the proximal *IL8* promoter, we cloned 500 bp of the endogenous *IL8* promoter and mutants with deletions of predicted AP-1 and NFAT binding sites to drive expression of nanoluciferase (Figure 5G). CPB-induced activation of the *IL8* promoter led to an approximately 1.5-fold increase in the luciferase activity of sheared THP-1 cells compared with the static cells (Figure 5H). When the binding site #1 for AP-1 or #2 for NFAT were deleted, there was no increase in the luciferase activity after shear (Figure 5H). These results confirmed that the binding of AP-1 and NFAT at these 2 adjacent binding sites activates the *IL8* transcription in THP-1 cells.

### CPB-induced monocytic cell death is partly mediated by TNF-α.

**Figure 6:**
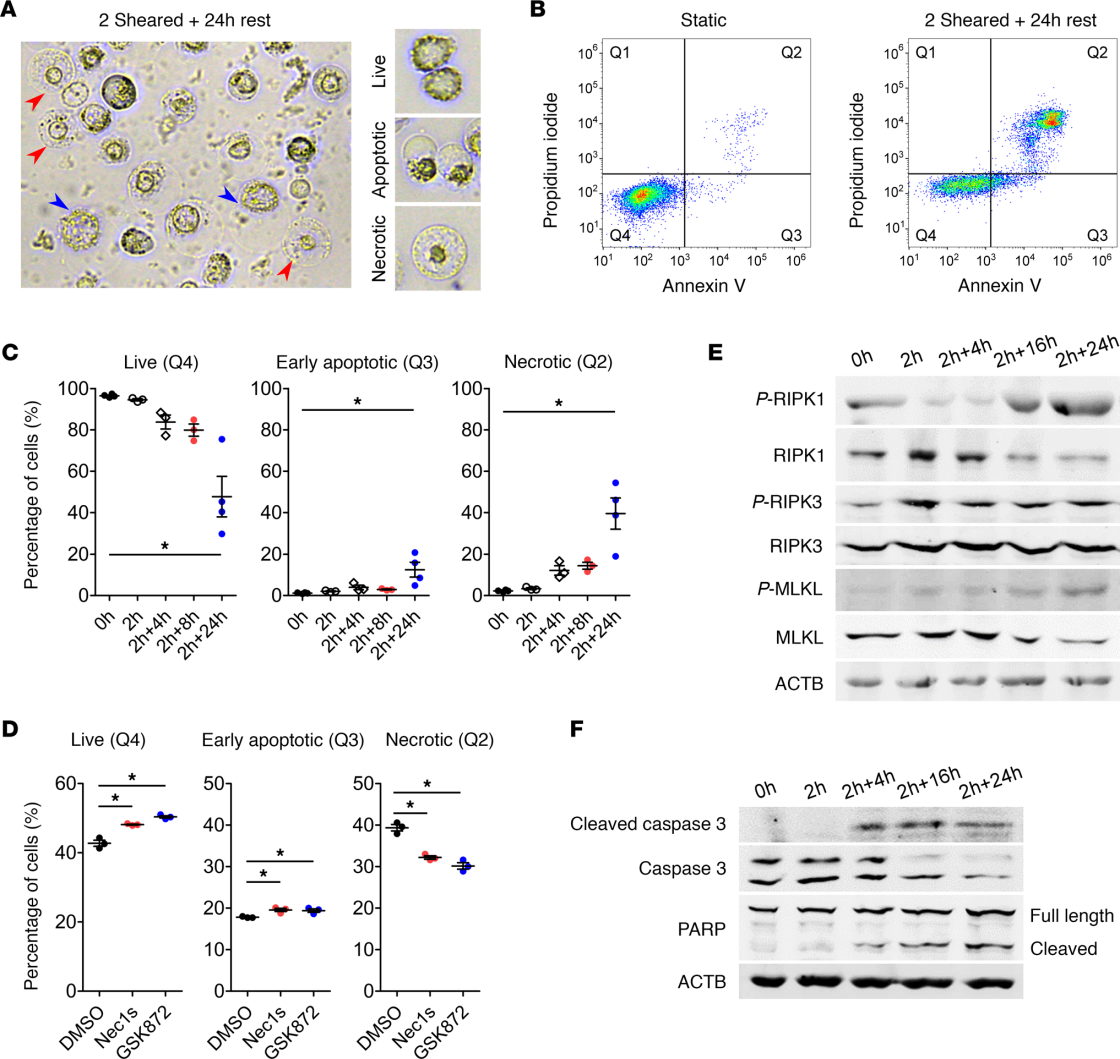
CPB induces cell death by both apoptosis and necroptosis. (**A**) Representative image of sheared THP-1 cells after 24 hours of recovery showed dead cells with morphology characteristic of both apoptosis (blue arrowheads) and necrosis (red arrowheads). (**B**) Sheared THP-1 cells were stained with annexin V and propidium iodide and were analyzed by flow cytometry. There was accumulation of dead cells in the Q2 quadrant after 24 hours compared with the static control. (**C**) The percentage of live cells (Q4) significantly reduced at 24 hours after shear, while the percentage of early apoptotic cells (Q3) or necrotic cells (Q2) significantly increased at 24 hours after shear (*n* = 3–4 replicates/group). (**D**) Treatment of sheared THP-1 cells with the necroptosis inhibitors Nec1s and GSK872 significantly increased the percentage of live cells (Q4) and early apoptotic cells (Q3), while significantly reducing the percentage of necrotic cells (Q2) (*n* = 3 replicates/group). (**E** and **F**) Western blot showed increased levels of necroptotic markers p-RIPK1, p-RIPK3, and p-MLKL (**E**) and apoptotic markers cleaved caspase-3 and cleaved PARP (**F**) in sheared THP-1 cells over time. *P < 0.05, 1-way ANOVA and post hoc Dunnett’s test.

We noticed that, immediately after being exposed to the in vitro CPB system for 2 hours, approximately 1.6% of the THP-1 cells were ruptured (Supplemental Figure 5A), likely due to mechanical disruption. When the remaining sheared cells were allowed to recover in the static condition, expression of the adhesion and migration marker intracellular adhesion molecule 1 (*ICAM1*) increased as early as 4 hours of recovery (Supplemental Figure 5B), suggesting that the cells had increased capability to migrate transendothelially into tissues. After 24 hours of recovery, approximately 24.7% of the cells became adherent to the plate and showed morphology characteristics of macrophages (Supplemental Figure 5C). Interestingly, a significant population of the nonadherent THP-1 cells died over time with morphology characteristics of both apoptotic and necrotic cell death (Figure 6A). To study this population, we stained sheared THP-1 cells that remained in suspension with annexin V and propidium iodide and analyzed them by flow cytometry for accumulation of cells in each quadrant (Figure 6B). The population of live cells in quadrant 4 (Q4) significantly decreased over time, while the population of early apoptotic cells in Q3 increased to approximately 13% after 24 hours (Figure 6C). Necrotic cells in Q2 are indicative of either late apoptotic, primary necrotic, or secondary necrotic cells, and this population significantly increased to approximately 40% after 24 hours (Figure 6C; the final percentages of all populations are in Supplemental Figure 5D). To examine whether cells in Q2 are primary necrotic, we treated sheared THP-1 cells with Necrostatin 1 (Nec1s) and GSK872, chemical inhibitors of necroptosis, and we found that they could indeed significantly reduce the Q2 population (Figure 6D). Western blot analysis showed increased levels of critical proteins in the necroptotic pathway (p-RIPK1, p-RIPK3, and p-MLKL) in the sheared THP-1 cells over time (Figure 6E). The levels of apoptotic markers: cleaved caspase-3 and cleaved PARP also increased in sheared THP-1 cells (Figure 6F). The level of active caspase-1, the mediator of pyroptosis, was not altered in sheared THP-1 cells (Supplemental Figure 5E). This result indicated that THP-1 cells died by both apoptosis and necroptosis after CPB. We attempted to quantify the proportions of apoptosis and necroptosis based on the cellular morphology and found them to be approximately 12% and approximately 23%, respectively, in sheared THP-1 cells after 24 hours of recovery (Supplemental Figure 5F). No cell death was observed in the adherent differentiated THP-1 population (Supplemental Figure 5G). Furthermore, when human primary CD14^+^ monocytes were subjected to the in vitro CPB system, the cells showed significant cell death within 3–4 hours of recovery (Supplemental Figure 6A). Western blot analysis demonstrated increased levels of cleaved caspase-3 and p-RIPK1 in sheared monocytes compared with the static cells (Supplemental Figure 6B), corroborating the results from THP-1 cells. Finally, cleaved caspase-3 levels in total leukocytes did not change between sham and CPB piglets; however, p-RIPK3 levels trended higher in the CPB piglets after surgery as compared with the sham piglets (Supplemental Figure 6, C and D).

**Figure 7:**
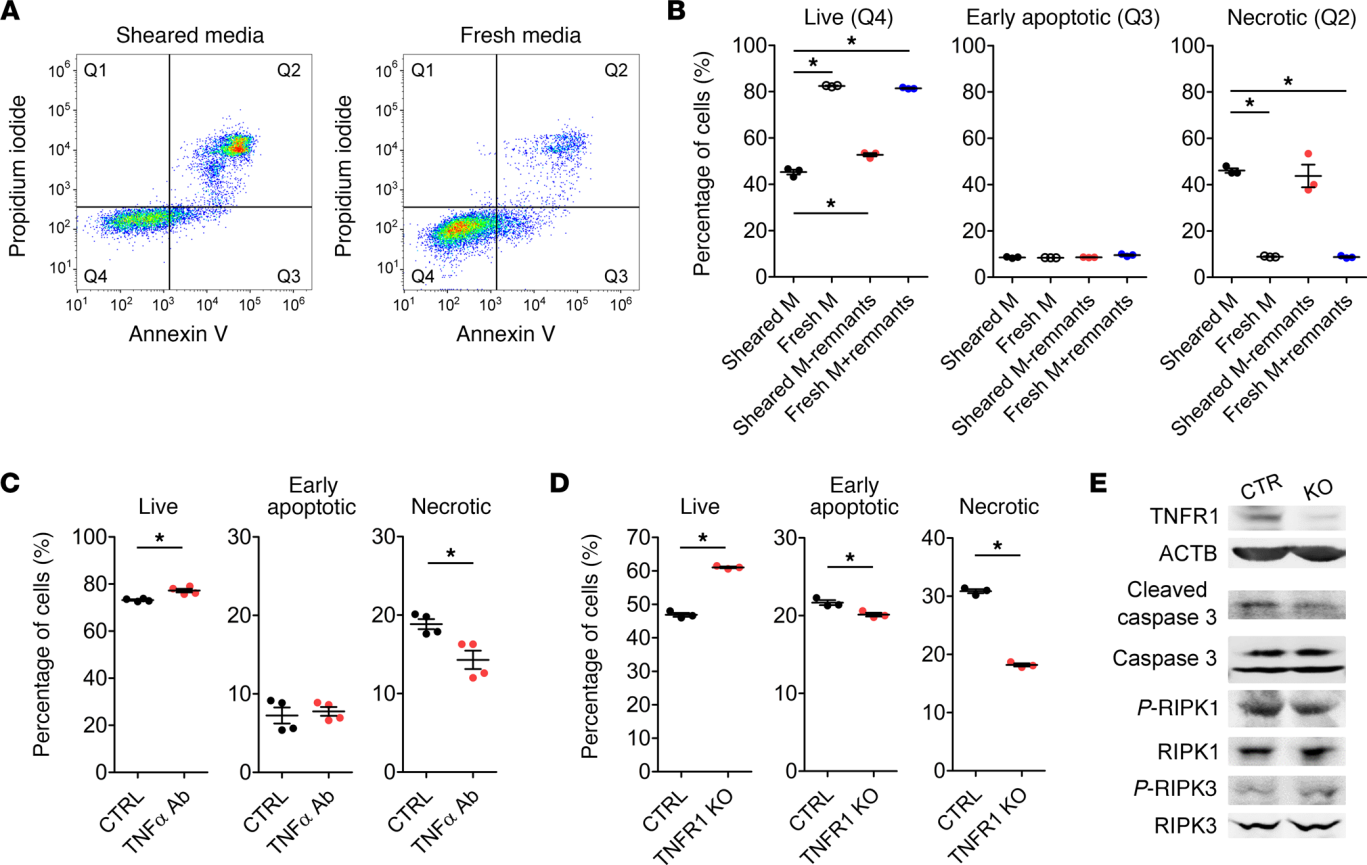
CPB-induced necroptosis is partly mediated by TNF-α. THP-1 cells were subjected to CPB conditions for 2 hours, recovered for 24 hours, and cell death was analyzed by flow cytometry. (**A** and **B**) When sheared THP-1 cells were changed to fresh media, the percentage of live THP-1 cells significantly increased, while the percentage of necrotic cells significantly decreased. Removing insoluble remnants of ruptured cells from the sheared media was not sufficient to rescue the cell death, while adding the remnants to the fresh media did not change the percentage of live or necrotic THP-1 cells (*n* = 3 replicates/group). (**C**) Treatment of sheared THP-1 cells with humanized TNF-α neutralizing antibody significantly increased the percentage of live cells and reduced the percentages of both early apoptotic and necrotic cells (*n* = 3 replicates/group). (**D**) CRISPR/Cas9-mediated knocking out of TNFR1 receptor significantly increased the percentage of live cells and reduced the percentages of both early apoptotic and necrotic cells compared with the control cells (*n* = 3 replicates/group). (**E**) Western blot showed reduction of cleaved caspase-3 and p-RIPK1 in sheared TNFR1-KO cells compared with control cells. These blots were run in parallel and contemporaneously on separate gels. *P < 0.05, 1-way ANOVA and post hoc Dunnett’s test (**B**), 2-tailed Student’s *t* test (**C** and **D**).

We then investigated if the cell death is precisely mediated by druggable factors/signaling pathways or merely an unavoidable consequence of mechanical insults. After 2 hours of being exposed to CPB condition, THP-1 cells were changed to fresh media, and strikingly, the cell death could be rescued; the percentage of live cells almost doubled, while the percentage of necrotic cells drastically reduced (Figure 7, A and B). We hypothesize that either insoluble remnants of the ruptured cells or soluble factors in the sheared media causes the cell death. Sheared media was then centrifuged at 20,000*g* for 10 minutes at room temperature to pellet the remnants. Removing the remnants from the sheared media or adding the remnants to the fresh media did not alter the percentage of cell death (Figure 7B). This result strongly indicates that the soluble factors released by sheared THP-1 cells into the supernatant caused cell death. Indeed, naive static THP-1 cells treated with the sheared media, with and without the remnants, experienced a marked increase in cell death (Supplemental Figure 7, A and B).

Since TNF-α is a known mediator of cell death, we examined if targeting soluble TNF-α could reduce necroptosis. Sheared THP-1 cells treated with the humanized TNF-α antibody, equivalent to adalimumab (Humira), experienced approximately 24% less necrotic cell death compared with the cells treated with the isotype control antibody (Figure 7C). CRISPR/Cas9-mediated KO of TNFR1, the receptor mediating cellular effects of TNF-α, reduced necrotic cell death by approximately 41% (Figure 7D) and decreased cleaved caspase-3 and p-RIPK1 levels (Figure 7E). These data demonstrate that cell death was partly mediated by TNF-α. Furthermore, when naive static THP-1 cells were treated with human recombinant IL-8, TNF-α, or both for 24 hours, the combination of IL-8 and TNF-α induced more cell death than TNF-α treatment alone, as shown by the cellular morphology (Supplemental Figure 7C) and confirmed by the increased levels of cleaved caspase-3, p-RIPK1, and p-RIPK3 (Supplemental Figure 7D). This suggests that, although IL-8 does not induce cell death directly, it might synergize with TNF-α to drive cell death.

## Discussion

This study is the first to our knowledge to profile global transcriptional changes in circulating leukocytes from neonatal patients during and after CPB. We delineated that myeloid cells are the major cell type driving the overall transcriptional response to CPB. The minimal involvement of lymphocytes could be attributed to their immaturity or that their response is delayed more than 24 hours after CPB. Our pathway enrichment analysis showed strong activation of myeloid cells for proinflammatory cytokine release, followed by a slightly delayed increase in antiinflammatory cytokine signaling. The balance between these 2 responses of the inflammatory network is believed to play a critical role in determining clinical outcomes (10). We also found that activation of TLR signaling in myeloid cells was initially suppressed but later increased at 8 hours after surgery, in agreement with a previous report (21). Other major pathways activated by CPB included early cell proliferation pathways, likely due to the margination of leukocytes and their precursors from bone marrow (22), and after CPB leukocyte migration as reported in the literature (15, 16).

Results from our CPB patient samples, piglet samples, and in vitro sheared THP-1 cells all showed that IL-8 and TNF-α are the main cytokines being directly upregulated by CPB. These data serve as confirmatory evidence that the inflammatory response is specific to the CPB procedure itself, without discounting the potential role of patients’ existing conditions, blood transfusion, and additives in exacerbating the inflammation. High shear stress was determined to be the most potent inducer of cytokine release. Since the flow patterns in the CPB circuit and our in vitro model are complex, further study is necessary to determine the critical shear patterns and thresholds that are required for the shear-mediated activation of leukocytes. The effect of plastic exposure could be overestimated in our study, as the PVC tubing we used is not coated, unlike the heparin-bonded circuits that have been shown to reduce cytokine release in patients (23). Moreover, we showed that hypothermia at 30°C, followed by rapid rewarming, exacerbates the inflammatory response in monocytes. In the context of CPB, however, the effect of temperature on cytokine release remains unclear. Warm CPB at 37°C was shown to upregulate TNF-α, IL-6, and IL-1β to a greater extent compared with cold CPB at 28°C–30°C in one study (24), while the levels of IL-8 and neutrophil elastase were found decreased in CPB at 34°C compared with hypothermic CPB at 28°C in another study (25). Since the readouts in these studies were not comparable, and the temperature effect in CPB is more complex than in our in vitro model, a more comprehensive study is warranted to conclude the benefit of hypothermic versus normothermic CPB for the inflammatory response.

The calcium chelator, EGTA, has previously been shown to prevent morphological changes caused by prolonged steady shear stress in neutrophils (26). In our study, extracellular calcium was indeed found essential for the CPB-induced upregulation of IL-8 and TNF-α, and MEK/ERK/AP-1 and CaN/NFAT were the major pathways involved. This conclusion from the in vitro CPB model agrees with our mRNA-Seq analysis from pediatric patient samples, which predicted the MEK/ERK pathway as being strongly activated and responsible for the transcriptional response to CPB. The MEK/ERK/AP-1 pathway has been widely established to drive expression of *IL8* in many different contexts (27), and the most common binding site of AP-1 is position –120 to –127 in the *IL8* promoter, which is site #3 in Figure 5G (27). Here, we demonstrated a different AP-1 binding site that is adjacent to a NFAT binding site as important for the shear stress–mediated activation of *IL8*, suggesting the 2 transcription factors interact synergistically. Inhibitors of MEK/ERK/AP-1 and CaN/NFAT pathways such as FK506 (Tacrolimus) could therefore be investigated for potential therapeutic effects to reduce systemic inflammation in pediatric CPB.

**Figure 8:**
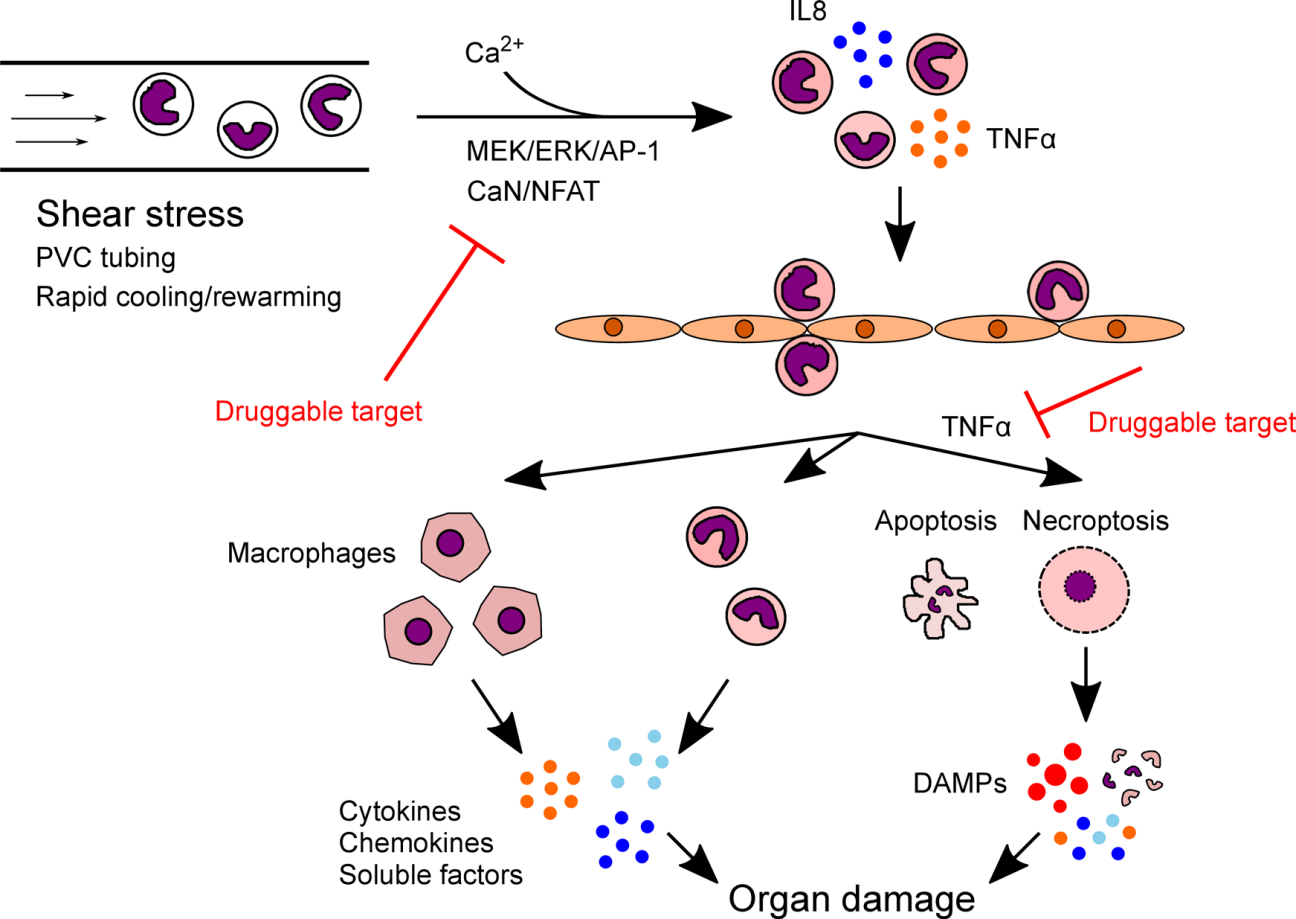
Proposed model for the mechanism of CPB-induced inflammation and organ damage. CPB conditions, particularly high level of shear stress, activate monocytes and upregulate production of inflammatory cytokines IL-8 and TNF-α via calcium-mediated MEK/ERK/AP-1 and CaN/NFAT pathways. Activated monocytes adhere and migrate through the vascular endothelium into peripheral organs, where they differentiate into macrophages over time. Macrophages, together with the remaining live inflammatory monocytes, secrete cytokines, chemokines, and damaging soluble factors into surrounding tissue environment. A subpopulation of activated monocytes dies by apoptosis and TNF-α–mediated necroptosis. Necroptotic monocytes burst and release DAMPs to further damage the organs. Targeting MEK/ERK/AP-1 and CaN/NFAT pathways to reduce monocyte activation or neutralizing TNF-α to prevent necroptosis could ameliorate post-CPB organ damage.

The mechanism of how systemic inflammation leads to organ damage is unclear. Evidence of CPB-activated leukocytes transmigrating and infiltrating into tissues is substantial in the literature (14–16), and our results further corroborate that CPB-activated THP-1 cells rapidly upregulated adhesion and migration markers after shear. Approximately 24.7% of the sheared monocytes later differentiated into macrophages; together with the live inflammatory monocytes, they secrete cytokines, chemokines, and other damaging soluble factors to the surrounding tissues. Uniquely, we found that approximately 26.6% of the sheared monocytes died via both apoptosis and necroptosis, the latter of which could release DAMPs leading to significant tissue injuries (Figure 8). This is the first study to our knowledge to demonstrate that CPB and its associated shear stress is sufficient to activate necroptosis and RIPK signaling. Furthermore, the cell death was found not due to mere mechanical disruption, but precisely mediated by soluble factors released in the supernatant. Neutralizing TNF-α, a recognized mediator of both apoptosis and necroptosis pathways (19, 28), appeared to be promising to reduce cell death and, potentially, subsequent tissue damage.

Neutrophils are the major type of myeloid cells that were not included in our study. The fact that neutrophils are directly exposed to CPB conditions while concurrently being activated by monocyte-derived IL-8 complicates the interpretation of the response; hence, it requires a different experimental setup in future studies. We have also not investigated the interactions of monocytes with other cell types such as the endothelium and platelets, which are both crucial for the pathogenesis of organ damage after CPB. Nevertheless, our study brought us a step closer to mechanistically deconvoluting the complex systemic inflammatory response in CPB by demonstrating the crucial roles of monocyte activation in systemic inflammation and the inflammatory monocytic cell death in tissue injuries. Besides the translational potential, our study provides a potentially novel understanding of basic biology of circulating leukocytes in response to fluid shear stress. Understanding the impacts of high biomechanical forces on circulating leukocytes could have broad applications to other diseases, such as aortic valve stenosis, or in the context of extracorporeal membrane oxygenation and ventricular assist devices.

## Methods

Supplemental Methods are available online with this article.

### Human blood samples.

Pediatric patients less than 1 month old with different congenital heart defects requiring repair utilizing CPB were enrolled in our study at Mercy’s Children Hospital (Kansas City, Missouri, USA). Written informed consent was received from participants’ parents or legal guardians prior to inclusion in the study. A standard CPB protocol was utilized in all of the patients. Approach in all cases was via median sternotomy. Aortic cannulation was used for the arterial access, with either single venous cannula being placed in the right atrium or bicaval cannulation, depending on the type of the defect requiring repair. The CPB circuit was blood primed, and standard additives included Solu Medrol, sodium bicarbonate, cefazolin, and tranexamic acid. After initiation of CPB, the patients were cooled to 18°C–30°C. Mean perfusion pressure was maintained at 25–35 mmHg, and the minimum hematocrit was kept at 24%. Upon completion of repair, the patients were rewarmed and weaned from CPB. MUF was performed in all of the patients after weaning from CPB. Blood samples of 2.5 mL were collected from an indwelling patient line or from the CPB pump, dependent upon the time of the blood draw (for blood draws performed while the patient was on CPB, these samples were pulled from the CPB pump). These samples were collected in EDTA tubes at 7 time points: before CPB (CPB-0h), 1 hour into CPB (CPB-1h), end of CPB (CPB-end), end of MUF (MUF-end), and 1 hour (MUF-1h), 8 hours (MUF-8h), 24 hours (MUF-24h) during postoperative recovery. Blood was centrifuged at 1000*g* for 20 minutes at room temperature, and plasma was removed. RBC were lysed in RNase-free RBC lysis solution (PerfectPure RNA blood kit, 5Prime) for 5 minutes at room temperature. The tubes were centrifuged at 2000*g* for 5 minutes at room temperature. The pellets of nucleated cells were lysed in the cell lysis buffer and immediately stored at –80°C till RNA extraction. The design and time course of the study are illustrated in Figure 1A. Patient demographics are in Supplemental Table 1.

### Whole transcriptome shotgun sequencing.

Total RNA from the nucleated cells was extracted using either the MirVana RNA extraction kit (Invitrogen) or PAXgene Blood RNA Kit (Qiagen). Illumina TruSeq RNA Libraries were prepared from 1 μg total RNA. High-throughput (6.2 GB), paired-end (2 × 101 bp), deep sequencing coverage (104×) RNA-Seq using Illumina’s TruSeq technology was performed on the Illumina HiSeq 1500 (CMH Genetics Research Core Lab). The mRNA-Seq data are available at the Gene Expression Omnibus under the dataset, GSE143780 (https://www.ncbi.nlm.nih.gov/geo/).

### Animal model.

Twelve male farm piglets from multiple litters were obtained from S&S Farms at 1 month old. Both sham and bypass groups (*n* = 6 piglets/group) received anesthesia, intubation, and median sternotomy. For the bypass group, animals were perfused with a CPB arterial pump flow rate of 100–120 mL/kg/min. Body temperature was cooled to 30°C. Fifteen to 20 minutes after CPB initiation, the piglets received 1-hour cardiac arrest with the ascending aortic clamp and cold del Nido cardioplegia solution (20 mL/kg). Body temperature was warmed to 37°C, and after 2 hours of total CPB support, perfusion flow of CPB was decreased gradually and CPB was then weaned. Six hours after CPB, animals were euthanized. Blood was collected at 5 time points: before median sternotomy (pre-CPB), 1 hour into CPB (CPB-1h), end of CPB (CPB-end), 1 hour after CPB (post-1h), and 6 hours after CPB (post-6h). Leukocytes were extracted and lysed in either Trizol for RNA isolation or RIPA buffer for protein analysis.

### Cell culture and treatments.

For the in vitro CPB model, monocytes at a density of 2 million cells/mL were sheared in a 10 foot-long Masterflex Tygon E-3603 L/S13 pump tubing (Cole-Parmer), using the Masterflex miniflex pump model 115/230 VAC 07525-20 (Cole-Parmer) at 10 mL/min. The majority of the tubing was submerged in the water bath for temperature control at either 30°C or 37°C. The design is illustrated in Figure 3A. In experiments studying the effect of extracellular calcium, THP-1 cells were washed twice with PBS and changed to either RPMI-1640 media supplemented with 5 mM EGTA (pH adjusted to 7.4) or calcium-free, serum-free DMEM media (21068028, Thermo Fisher Scientific) right before shearing; the full RPMI-1640 media mentioned above or the matching calcium-containing serum-free DMEM media (11960044, Thermo Fisher Scientific) were used as the control media respectively.

For CRISPR/Cas9-mediated gene KO, we first generated a doxycycline-inducible Cas9-expressing THP-1 cell line (iCas9-expressing cells) by transducing THP-1 cells with lentiviruses carrying the Lenti-iCas9-neo plasmid (a gift from Qin Yan; Addgene plasmid no. 85400). Multiple-guide RNAs targeting a gene were cloned into the Lenti-multi-Guide plasmid (a gift from Qin Yan; Addgene plasmid no. 85401) and delivered to iCas9-expressing THP-1 cells by the lentiviral system. Cells were then treated with 1 μg/mL of doxycycline for 48 hours to induce editing at the targeted loci. The sequences of the gRNAs targeting TNFR1 are 5′-ATTGGACTGGTCCCTCACCT-3′, 5′-AGAGGTGCACGGTCCCATTG-3′, 5′-GTACAATGACTGTCCAGGCC-3′.

### Luciferase reporter assay.

A total of 500 bp of the *IL8* promoter and mutants with deletions of different predicted NFAT and AP-1 binding sites was cloned to replace the CMV promoter in the pCDH-CMV-NLuc plasmid (a gift from Kazuhiro Oka; Addgene plasmid no. 73038). THP-1 cells were transduced with lentiviruses carrying different promoter reporter plasmids. Each cell line was sheared for 2 hours at 30°C, and after 5 hours of recovery in the incubator, cells were lysed and the activity of NanoLuc luciferase was determined using the Nano-Glo Luciferase assay system (Promega). The signals were normalized to the protein content of the samples.

### Statistics.

For RNA-Seq, reads were aligned to the GRCh38/hg38 version of the human genome using STAR, and only uniquely alignable reads (MAPQ > 10) were kept for downstream analysis. Gene expression levels were calculated by counting reads whose 5′ ends overlapped with RefSeq-defined exons in a strand specific manner, using HOMER’s command analyzeRepeats.pl, allowing reads to be assigned to more than 1 transcript if the exons overlap. For genes with multiple isoforms as assigned by their Entrez Gene IDs, the isoform with the highest number of reads was selected as representative for the gene, and the others were discarded. Raw, unnormalized read counts for genes in each sample were then analyzed using DESeq2 using a design matrix that accounts for samples from the same patient (i.e., paired analysis).

Evaluation of gene expression patterns across time points was performed by hierarchical clustering of DE genes based on Euclidean distances using gplots in R to identify unique expression clusters. Analysis of gene expression kinetics was performed using tidyverse in R. Gene ontology and biological pathway analysis was performed using Metascape (29) and Ingenuity Pathway Analysis (IPA) (30). Analysis of cell type–specific gene set enrichment was performed using the Gene Atlas database in EnrichR (31). Inferences of the activation state of upstream regulatory kinases were performed using IPA (30).

All other statistical analyses were performed using Prism 5 (GraphPad). Numeric differences between 2 groups were compared using a 2-tailed Student’s *t* test. Comparisons of experimental groups against a control group (e.g., CPB-0h) were performed using 1-way ANOVA and post hoc Dunnett’s test. Other pairwise comparisons for more than 2 groups were performed using 1-way ANOVA and post hoc Tukey’s test. Human and piglet data that did not pass the Kolmogorov-Smirnov test for normal distribution and Bartlett’s test for equal variances were analyzed using Kruskal-Wallis test instead of 1-way ANOVA. *P* < 0.05 was considered significant. Data are represented as mean ± SEM, and replicates are as indicated in figure legends.

### Study approval.

Human blood samples were collected from pediatric patients undergoing CPB surgery under an IRB at Children’s Mercy Hospital, Kansas City (IRB protocol no. 14110493) and Institutional Biosafety Committee at University of Missouri, Kansas City (IBC protocol no. 17-35). Animal use in this study was approved by the IACUC of the Seattle Children’s Research Institute and adhered to the *Guide for the Care and Use of Laboratory Animals* (National Academies Press, 2011).

## Author contributions

LNT and VN conceived the ideas, designed the experiments, and wrote the manuscript. LNT conducted most of the experiments and acquired and analyzed the data. LH and SH conducted some in vitro experiments. MK, KC, MAP, and MN performed the piglet CPB surgery. NK, JAM, JOB, DB, and PP collected human blood samples and isolated total mRNAs for whole transcriptome sequencing. CB, AF, and RS performed statistical analysis for the mRNA-Seq data. AA and MS helped design the in vitro CPB model.

## Supplementary Material

Supplemental data

## Version 1 11/24/2020

In-Press Preview

## Version 2 01/11/2021

Electronic publication

## Version 3 04/21/2021

The spelling of Peter Pastuszko's name was corrected.
