# Supplementary material for: Shear stress associated with cardiopulmonary bypass induces expression of inflammatory cytokines and necroptosis in monocytes
Source: JCI Insight. 2021 Jan 11;6(1):e141341. doi: 10.1172/jci.insight.141341 (PMC7821587; doi:10.1172/jci.insight.141341)
Supplement: Supplemental data [file jciinsight-6-141341-s009.pdf]

Expanded Materials and Methods

Whole transcriptome shotgun sequencing

Total RNA from the nucleated cells was extracted using either the MirVana RNA extraction kit (Invitrogen) or PAXgene Blood RNA Kit (Qiagen). The concentration and quality of total RNA for each sample were quantified and evaluated by spectrophotometric (Epoch; Thermo Scientific) and automated chip electrophoresis analyses (Experion, BioRad) respectively. Each sample met the quality standards of a 260/280 ratio > 2.0 and a RNA integrity number (RIN) > 9. Illumina TruSeq RNA Libraries were prepared from 1 µg total RNA.

High throughput (6.2GB), paired-end (2 x 101), deep sequencing coverage (104X) RNA-seq using Illumina's TruSeq technology was performed on the Illumina HiSeq 1500 (CMH Genetics Research Core Lab). The mRNA-Sequencing data is available at the Gene Expression Omnibus under the dataset, GSE143780.

Animal model

12 male farm piglets from multiple litters were obtained from S&S Farms (California, USA) at 1-month old. Piglets were initially sedated with an intramuscular injection of ketamine (33 mg/kg) and xylazine (2 mg/kg), and were placed on a circulating warming blanket to keep a rectal temperature of 36-37.5°C. After intubation through surgical tracheostomy, the piglets were mechanically ventilated with an oxygen (40-50%) and isoflurane (1-2%) mixture in a monitored setting of electrocardiogram and oxygen saturation. A PowerLab 16/30 recorder (AD Instruments, Colorado Springs, CO) was used to continuously record hemodynamic data throughout surgical procedure. Angiographic catheters were inserted into the right femoral artery and central vein through the left external jugular vein for continuous recording of the pressure, fluid infusion and collection of blood samples. Surgical skin preparation was done with povidone iodine. After median sternotomy, a flow probe (Transonic Systems Inc, Ithaca, NY) around the ascending aorta was attached, and left ventricular pressure catheter (Millar Instruments, Houston, TX) was directly inserted. All animals, both sham and bypass groups (n=6 piglets/group) received the above treatment.

Specifically for the bypass group, cardiopulmonary bypass (CPB) with a roller peristaltic pump console (Stockert S3) and a hollow fiber membrane oxygenator (CX-RX05RW, Terumo, Tokyo, Japan) was established by central cannulation via the ascending aorta (12-14 Fr) and right atrium (20-24 Fr) after systemic heparinization (300 IU/kg, ACT > 400 sec). Animals were perfused with a CPB arterial pump flow rate of 100-120 mL/kg/min. The piglets were maintained at a pH of 7.35 to 7.45, an arterial pCO_2 of 35 to 45 mmHg and an arterial pO_2 of greater than 120 mmHg. Body temperature was slowly cooled down to 30°C. 15-20 mins after CPB initiation, the piglets received 1-hour cardiac arrest at 30°C with the ascending aortic clamp and cold del Nido cardioplegia solution (20 mL/kg). Blood was continuously concentrated using a hemoconcentrator (HPH Junior, Minntech). Body temperature was warmed up to 37°C, and after 2 hrs of total CPB support, perfusion flow of CPB was decreased gradually and CPB was then weaned. Epinephrine was given if necessary. The aortic and venous cannulas were removed.

The chests of both sham and bypass piglets remained open while recovering under anesthesia and received Normosol R for fluid infusion. 6 hrs after CPB, the whole body was perfused with 3 L ice cold PBS and animals were euthanized. Throughout the experiment, 1 mL of blood was collected at 5 time points in a heparinized tube: before median sternotomy (pre-CPB), 1 hr into CPB (CPB-1h), end of CPB (CPB-end), 1 hr after CPB (Post-1h), and 6 hrs after CPB (Post-6h). Blood was centrifuged at 1,500g for 7 mins. Plasma was removed, the remaining cell pack volume

was diluted with 12 mL of RBC lysis buffer (eBioscience) and gently rocked for 10 mins at room temperature. The tubes were centrifuged at 500g for 5 mins, the pellets of leukocytes were further washed with 1 mL ice-cold PBS and then lysed in either Trizol for RNA isolation or radioimmunoprecipitation assay (RIPA) buffer for protein analysis.

Cell culture and treatments

Human monocytic cells THP-1 (ATCC) were cultured in RPMI-1640 media supplemented with 10% FBS (Gibco), 50 U/mL penicillin, and 50 µg/mL streptomycin (Gibco) in 5% CO_2 at 37°C. Human primary peripheral blood CD14+ monocytes (StemCell) were thawed according to the manufacturer's protocol in the same media, rested for 1 hr in 5% CO_2 at 37°C and used immediately for experiments. Human embryonic kidney cells Lenti-X 293T (Clontech) were cultured in DMEM media supplemented with 10% FBS (Gibco), 50 U/mL penicillin, and 50 µg/mL streptomycin (Gibco) in 5% CO_2 at 37°C.

For the in vitro CPB model, monocytes at a density of 2 million cells/mL were sheared in a 10ft-long Masterflex® Tygon E-3603 L/S13 pump tubing (Cole-Parmer), using the Masterflex® miniflex pump model 115/230 VAC 07525-20 (Cole-Parmer) at 10 mL/min. Majority of the tubing was submerged in the water bath for temperature control at either 30°C or 37°C. The design is illustrated in Fig 3A. In experiments studying the effect of extracellular calcium, THP-1 cells were washed twice with PBS and changed to either RPMI-1640 media supplemented with 5 mM EGTA (pH adjusted to 7.4) or calcium-free, serum-free DMEM media (21068028, Gibco) right before shearing; the full RPMI-1640 media mentioned above or the matching calcium-containing serum-free DMEM media (11960044, Gibco) were used as the control media respectively. In some experiments, THP-1 cells were pre-incubated for 30 mins with either the vehicle or one of the following chemicals: 10 µM U0126 (Abcam), 30 µM FR180204 (Cayman), 5 µM FK506 (Cayman), 10 µM INCA-6 (Abcam), 5 µM GO 6983 (Abcam), 5 µM Sotrastaurin (Cayman), 40 µM STO-609 (Abcam), 5 µM Calmodulin Kinase IINtide, myristoylated (EMD Millipore) before shearing.

For genetic modification, lentiviral vectors together with helper plasmids encoding gag, pol and rev, were co-transfected in 293T cells using TransIT-Lenti (Mirus Bio). Viral supernatants were collected at 48 hrs and 72 hrs after transfection and centrifuged at 1,000 g for 10 mins. THP-1 cells were then transduced at the multiplicity of infection of 8 using the viral supernatants supplemented with 5 µg/mL polybrene for 6 hrs. Three days after transduction, positive cells were selected in 1 µg/mL puromycin for 72 hrs, if necessary. Specifically for CRISPR/Cas9-mediated gene knockout, we first generated a doxycycline-inducible Cas9-expressing THP-1 cell line (iCas9-expressing cells) by transducing THP-1 cells with lentiviruses carrying the Lenti-iCas9-neo plasmid (a gift from Qin Yan; Addgene plasmid #85400). Multiple guide RNAs targeting a gene were cloned into the Lenti-multi-Guide plasmid (a gift from Qin Yan; Addgene plasmid #85401) and delivered to iCas9-expressing THP-1 cells by the lentiviral system. Cells were then treated with 1 µg/mL of doxycycline for 48 hrs to induce editing at the targeted loci. The sequences of the gRNAs targeting TNFR1 are 5' – ATTGGACTGGTCCCTCACCT – 3', 5' – AGAGGTGCACGGTCCCATTG – 3', 5' – GTACAATGACTGTCCAGGCC – 3'.

Immunofluorescence

THP-1 cells were fixed in 2% paraformaldehyde for 10 mins at room temperature, centrifuged at 1,000g for 5 mins and washed with 1 mL of PBS. Cells were suspended in 20 µL of PBS, spread on microscope slides, let air-dried and rinsed with water before being stored at 4°C. Immunofluorescence analyses were performed using antibodies against NFAT1 (5861, Cell Signaling), p-c-JUN (3270, Cell Signaling), NF-kB p65 (8242, Cell Signaling) and CREB-1 (sc-240, Santa Cruz). Semiquantitative analyses of cells double-positive for DAPI and other proteins were performed by ImageJ.

Western blot and ELISA

Total protein concentrations in cell lysates were measured using the bicinchoninic acid (BCA) assay (Thermo Fisher). 10-15 µg of proteins were separated by SDS-PAGE and immunoblotted using the following antibodies: p-ERK1/2 (9101, Cell Signaling), ERK1/2 (4695, Cell Signaling), p-c-JUN (3270, Cell Signaling), c-JUN (9165, Cell Signaling), p-IKKα/β (2078, Cell Signaling), IKKβ (2678, Cell Signaling), p-IkB-α (2859, Cell Signaling), p-RIPK1 (65746, Cell Signaling), RIPK1 (3493, Cell Signaling), p-MLKL (91689, Cell Signaling), MLKL (14993, Cell Signaling), cleaved caspase-3 (9664, Cell Signaling), caspase 3 (14220, Cell Signaling), PARP (9532, Cell Signaling), TNFR1 (3736, Cell Signaling), p-NFAT2 (MAB5640, Novus), NFAT2 (NB100-56732, Novus), p-NFAT1 (PA5-64484, Thermo Fisher), NFAT1 (5861, Cell Signaling), p-CREB (MAB6906, Novus), RIPK3 (MAB7604, Novus), CREB-1 (sc-240, Santa Cruz), p-CAMKII (sc-32289, Santa Cruz), CAMKII (sc-5306, Santa Cruz), IkB-α (sc-1643, Santa Cruz), p-RIPK3 (ab209384, Abcam), capase-1 (ab207802, Abcam) and the loading control β-actin ACTB (sc-47778, Santa Cruz). Simultaneous detection was performed using IRDye 700 and 800-labeled secondary antibodies (Li-Cor) under the Odyssey laser fluorescence scanner (Li-Cor) and quantified using ImageJ.

Supernatants of sheared monocytes were immediately frozen after collection. Quantification of cytokines was measured using the human ELISA Ready-SET-Go kits for IL1β, IL6, TNFα and IL8 (eBioscience) according to the manufacturer's protocol.

Quantitative PCR

Gene expressions were quantified using a SYBR® Green detection method (primer sequences in Supplemental Table S2). All expression data were normalized to internal control genes, 18S ribosomal RNA (*RN18S1*) and ribosomal protein L4 (*RPL4*) for human and pig samples, respectively. Relative quantification of fold-change was performed using the $2^{-\Delta/\Delta Ct}$ method.

Luciferase reporter assay

500bp of the *IL8* promoter and mutants with deletions of different predicted NFAT and AP-1 binding sites were cloned to replace the CMV promoter in the pCDH-CMV-NLuc plasmid (a gift from Kazuhiro Oka; Addgene plasmid #73038). THP-1 cells were transduced with lentiviruses carrying different promoter reporter plasmids. Each cell line was sheared for 2 hrs at 30°C and after 5 hrs of recovery in the incubator, cells were lysed and the activity of NanoLuc® luciferase was determined using the Nano-Glo® Luciferase assay system (Promega). The signals were normalized to the protein content of the samples.

Cell death and cell proliferation assays

THP-1 cells were stained with Alexa Fluor 488® annexin V and propidium iodide according to the manufacturer's protocol for the Dead cell apoptosis kit (V13241, ThermoFisher). Samples were immediately analyzed by flow cytometry. In some experiments, after 2 hrs of shearing, THP-1 cells were treated with the following chemicals for 24 hrs before analysis: 10 µM Nec-1s (Cayman), 10 µM GSK872 (Cayman), human IgG1 isotype control (0151L-01, Southern Biotech), Adalimumab-equivalent TNFα neutralizing antibody (MAB9677, Novus).

To quantify the number of THP-1 cells that adhered to the plate after shearing, both suspension and adhered fractions were washed with PBS and frozen-thawed for cell lysis. DNA content was measured using the CYQUANT® cell proliferation assay kit (ThermoFisher).

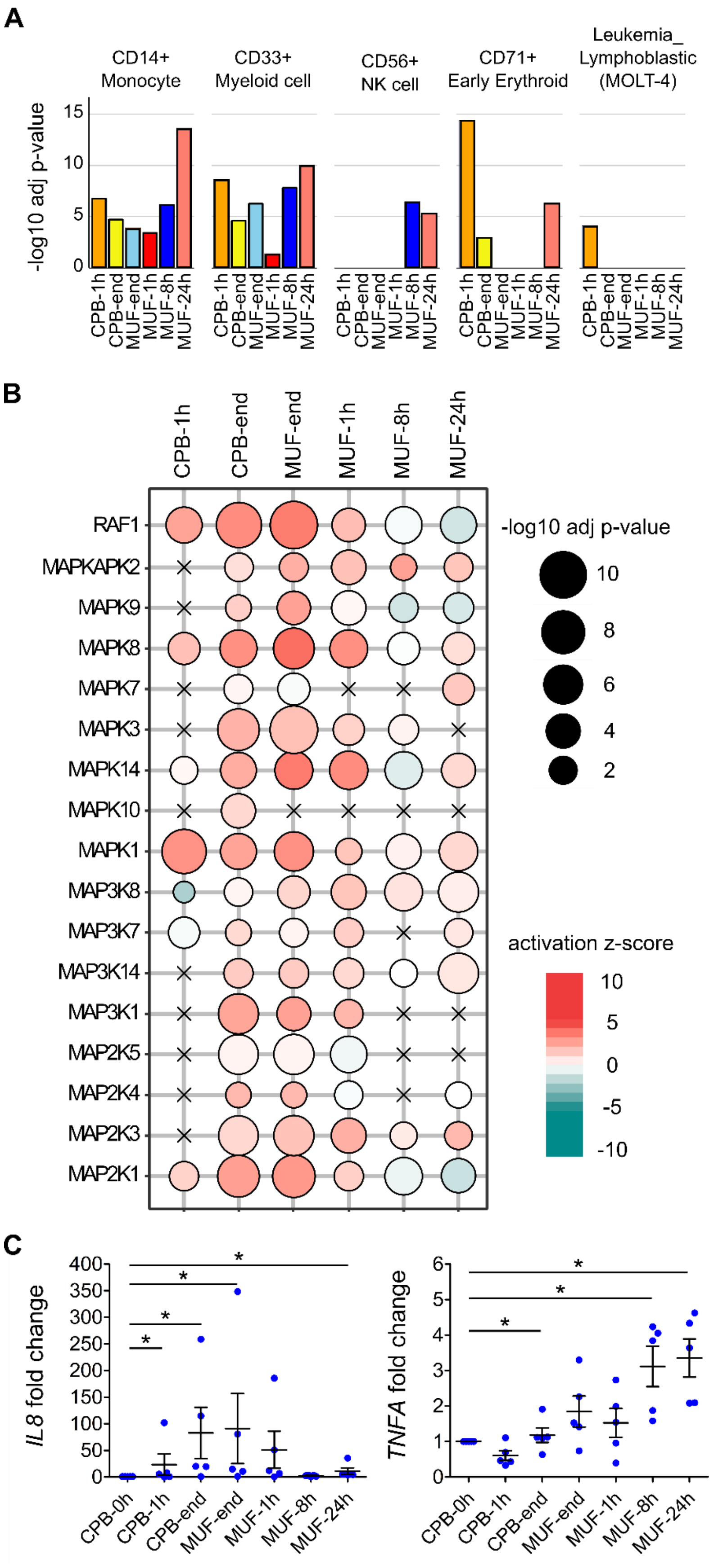
A
CD14+ Monocyte
CD33+ Myeloid cell
CD56+ NK cell
CD71+ Early Erythroid
Leukemia_ Lymphoblastic (MOLT-4)
-log10 adj p-value
15
10
5
0
CPB-1h
CPB-end
MUF-end
MUF-1h
MUF-8h
MUF-24h
B
CPB-1h
CPB-end
MUF-end
MUF-1h
MUF-8h
MUF-24h
RAF1
MAPKAPK2
MAPK9
MAPK8
MAPK7
MAPK3
MAPK14
MAPK10
MAPK1
MAP3K8
MAP3K7
MAP3K14
MAP3K1
MAP2K5
MAP2K4
MAP2K3
MAP2K1
-log10 adj p-value
10
8
6
4
2
activation z-score
10
5
0
-5
-10
C
IL8 fold change
400
350
300
250
200
150
100
50
0
CPB-0h
CPB-1h
CPB-end
MUF-end
MUF-1h
MUF-8h
MUF-24h
TNFA fold change
6
5
4
3
2
1
0

Figure S1. mRNA sequencing analysis of total nucleated cells from pediatric patients undergoing CPB. (A) Computational deconvolution of cell subsets across different time points using GeneAtlas in EnrichR. The bar graphs are the $-\log_{10}$ (adjusted p-value) enrichment scores ($-\log_{10}$ adjusted p-value > 1.3 was considered significant) for differentially expressed genes corresponding to distinct cell subsets. **(B)** Predicted activation state of MAP kinases with significantly associated transcriptional changes at each time point using Ingenuity Pathway Analysis (IPA). Bubble plot representation of significant enrichment scores (activation z-score > 2) in at least one time point. Color indicates predicted activation (red) or predicted inhibition (green) and bubble diameter represents the $-\log_{10}$ adjusted p-value as determined by Fisher's exact test. Crosses signify a lack of significant enrichment at specific time points. **(C)** mRNA-seq data analysis showed significant upregulation of *IL8* and *TNFA* during CPB and recovery (n=5 patients, *adjusted $p<0.05$).

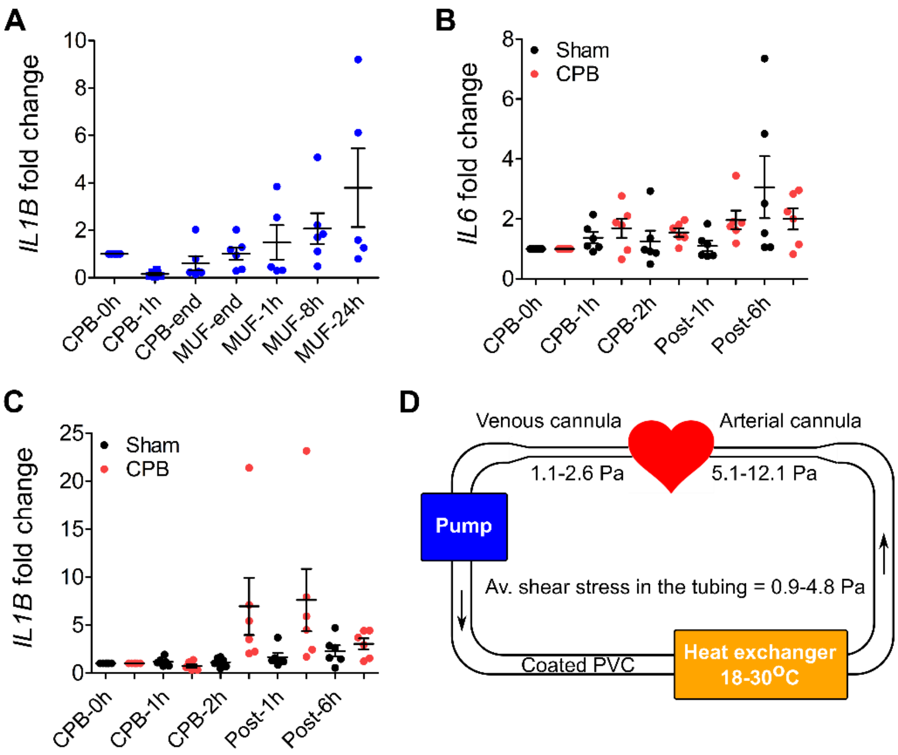

Figure S2. *IL1B* and *IL6* are not consistently upregulated in leukocytes by CPB condition. (A) Quantitative PCR analysis from total leukocytes isolated from pediatric patients undergoing CPB showed no changes in *IL1B* level after CPB compared to their own expression level before CPB (n=5-6 patients, an independent cohort). Kruskal-Wallis test. **(B-C)** Quantitative PCR analysis from total leukocytes isolated from the piglets showed no significant changes in *IL1B* and *IL6* levels in the CPB piglets compared to the sham controls (n=6 piglets/group). Kruskal-Wallis test. **(D)** Illustration of a typical CPB circuit used for pediatric patients or piglets less than 12 kg. The blood is pumped out of the heart via the venous cannula, through a long coated PVC tubing by either a roller or centrifugal pump. Temperature is maintained by the heat exchanger at 18-30°C depending on the case. Blood is returned to the body via the arterial cannula. The diameter of the PVC tubing varies based on the weight of the subject; the flow rate is continuously monitored and adjusted based on the weight of the subject, the cardiac output as well as other hemodynamic parameters. The average shear stress in the tubing therefore varies and is estimated ~0.9-4.8 Pa. The diameters of the cannulas are based on the weight of the subject and they are generally much smaller than the diameter of the tubing, causing the max shear stress for the blood cells passing through to reach ~1.1-2.6Pa and ~5.1-12.1 Pa for the venous and arterial cannulas respectively.

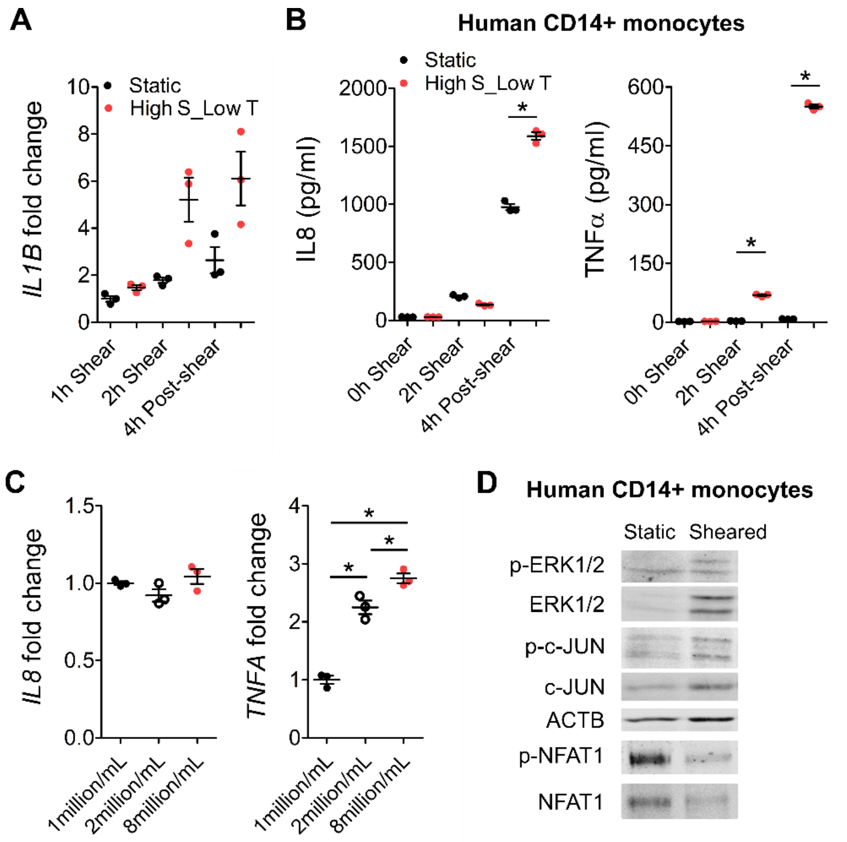

Figure S3. In vitro CPB condition specifically upregulates IL8 and TNFα in monocytes via MEK/ERK/AP-1 and CaN/NFAT pathways. (A) Quantitative PCR analysis showed that the CPB condition (High S_Low T) significantly upregulated the level of *IL1B* transcripts in sheared THP-1 cells compared to static cells after 2 hrs of shear (n=3 replicates/group). **(B)** Human primary CD14+ monocytes were subjected to in vitro CPB condition (High S_Low T). Secreted protein levels of IL8 and TNFα from sheared monocytes after 2 hrs of shear and after 4 hrs of recovery were significantly higher compared to those from static cells (n=3 replicates/group). **(C)** THP-1 cells at different cell densities were subjected to CPB condition. *IL8* level was not affected by the cell density, while *TNFA* level was significantly elevated with higher cell densities (n=3 replicates/group). **(D)** Western blot showed increased levels of p-ERK1/2 and p-c-JUN; and reduced level of p-NFAT1 in the human CD14+ monocytes after shear compared to the static controls. *$p<0.05$, one-way ANOVA and post hoc Tukey's test.

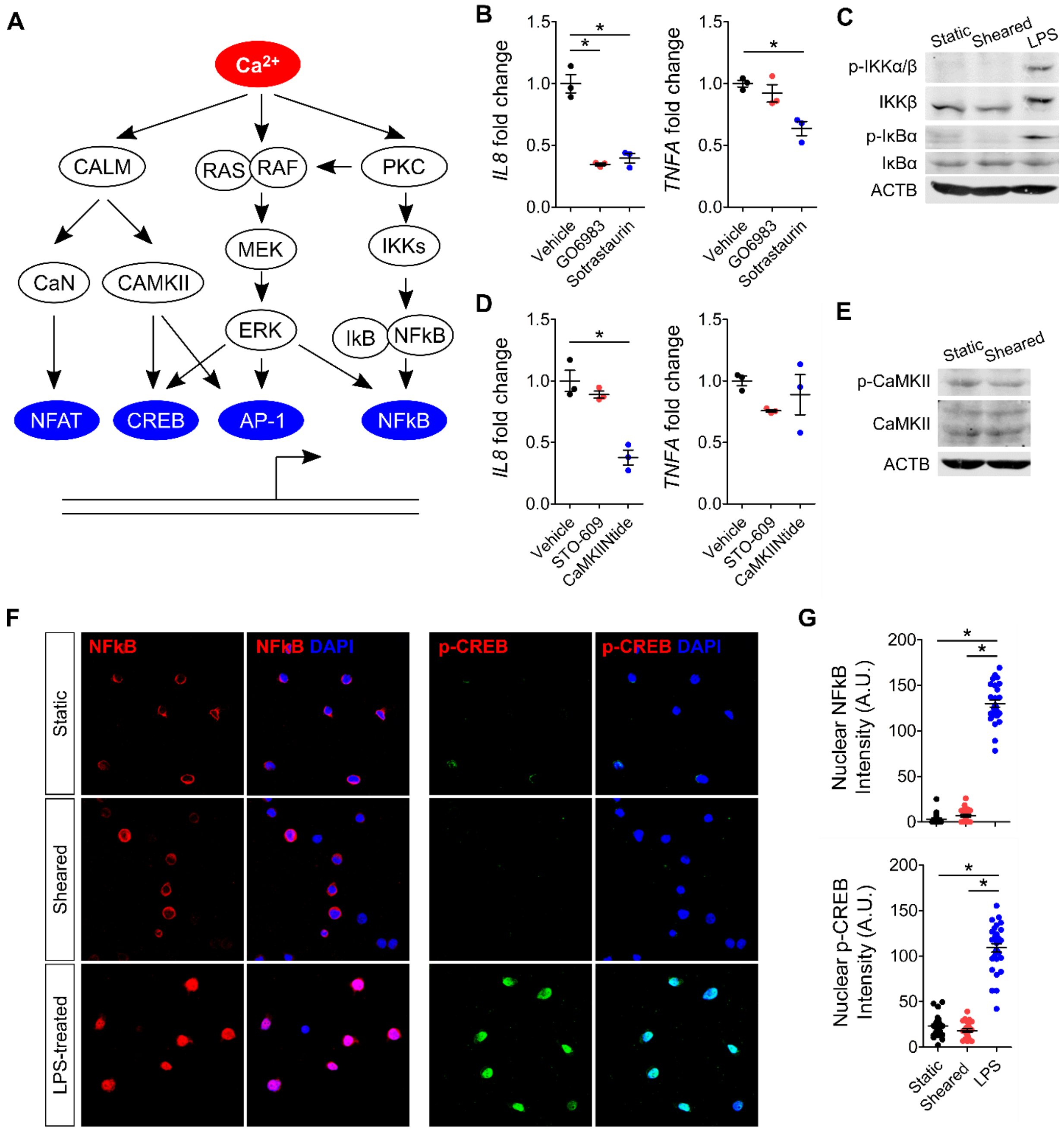

Figure S4. PKC/NFkB and CAMKII/CREB pathways are not involved in CPB-induced upregulation of *IL8* and *TNFA*. (A) Diagram showing calcium signaling pathways. **(B)** GO6983 and Sotrastaurin, inhibitors of PKC, significantly reduced upregulation of *IL8* after shear compared to the vehicle control (n=3 replicates/group). Upregulation of *TNFA* was significantly reduced with Sotrastaurin treatment but not with GO6983 (n=3 replicates/group). **(C)** Western blot showed no increase in levels of p-IKKα/β and p-IkBα in the sheared THP-1 cells compared to static cells. Lipopolysaccharides (LPS) treatment served as the positive control. **(D)** When THP-1 cells were treated with STO-609 and CAMKIINtide to inhibit function of CAMKII, upregulation of *IL8* after shear was significantly reduced compared to the vehicle control only with CAMKIItide treatment while upregulation of *TNFA* after shear was not affected by both treatments (n=3

replicates/group). **(E)** Western blot showed no change in the level of p-CAMKII in the sheared THP-1 cells compared to static cells. **(F-G)** Immunocytochemical analysis of the sheared THP-1 cells showed no expression of NFkB p65 or p-CREB in the nuclei. LPS treatment served as the positive control (n=23-30 cells/group). *$p<0.05$, one-way ANOVA and post hoc Dunnett's test (B,D), one-way ANOVA and post hoc Tukey's test (G).

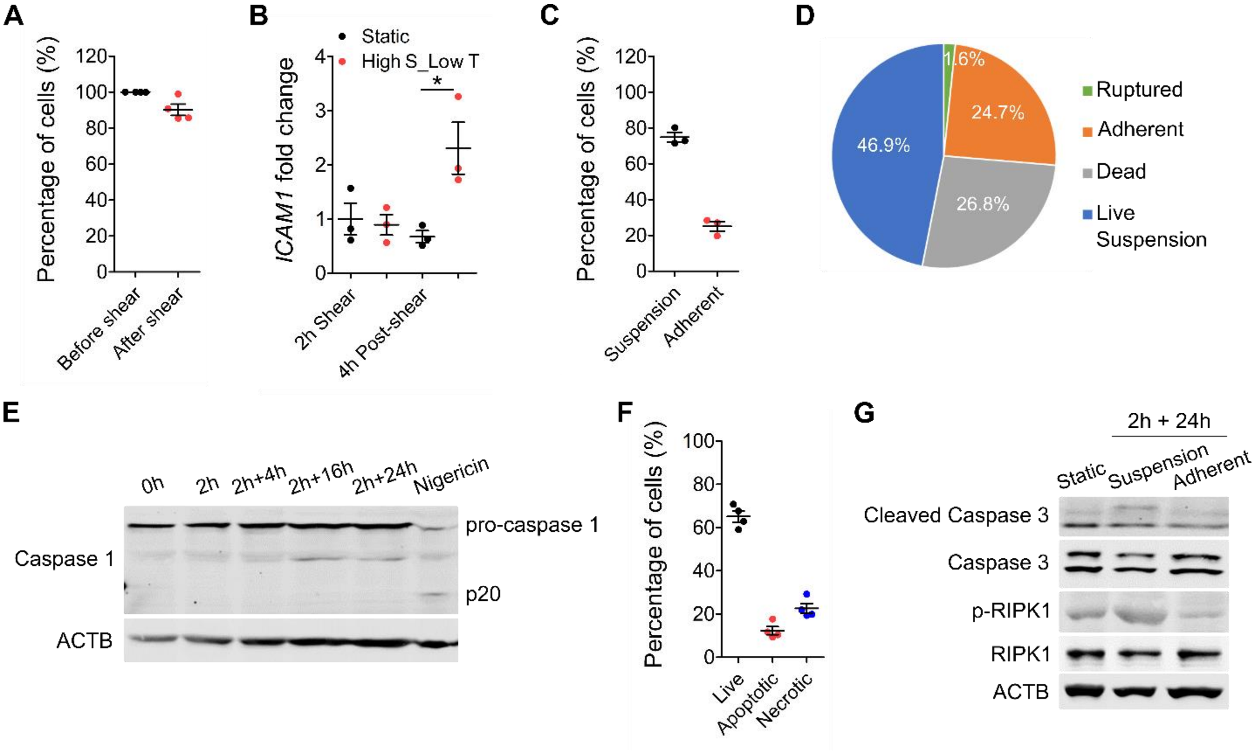

Figure S5. The fates of THP-1 cells after being exposed to CPB condition for 2 hrs. (A) Quantification of the number of THP-1 cells before and after shear showed that ~1.6% of cells were ruptured after being exposed to the in vitro CPB condition for 2 hrs (n=4 replicates/group). **(B)** Quantitative PCR analysis showed that after 4 hrs of recovery, the sheared THP-1 cells significantly upregulated *ICAM1* compared to the static control (n=3 replicates/group). *p<0.05, one-way ANOVA and post hoc Tukey's test. **(C)** After 24 hrs of recovery, ~24.7% of the sheared THP-1 cells became adherent (n=3 replicates/group). **(D)** Pie chart showing different populations of sheared THP-1 cells after 24 hrs of recovery. **(E)** Western blot showed no expression of cleaved caspase 1 (p20), the active form of the enzyme, in the sheared THP-1 cells over time. Nigericin treatment for 4 hrs served as the positive control. **(F)** Quantification of the percentage of live, apoptotic and necrotic THP-1 cells after 24 hrs of recovery based on the cellular morphology. **(G)** Western blot showed increased levels of apoptotic marker cleaved caspase 3 and necroptotic marker p-RIPK1 only in the suspension THP-1 cells and not in the adherent THP-1 cells after 24 hrs of recovery.

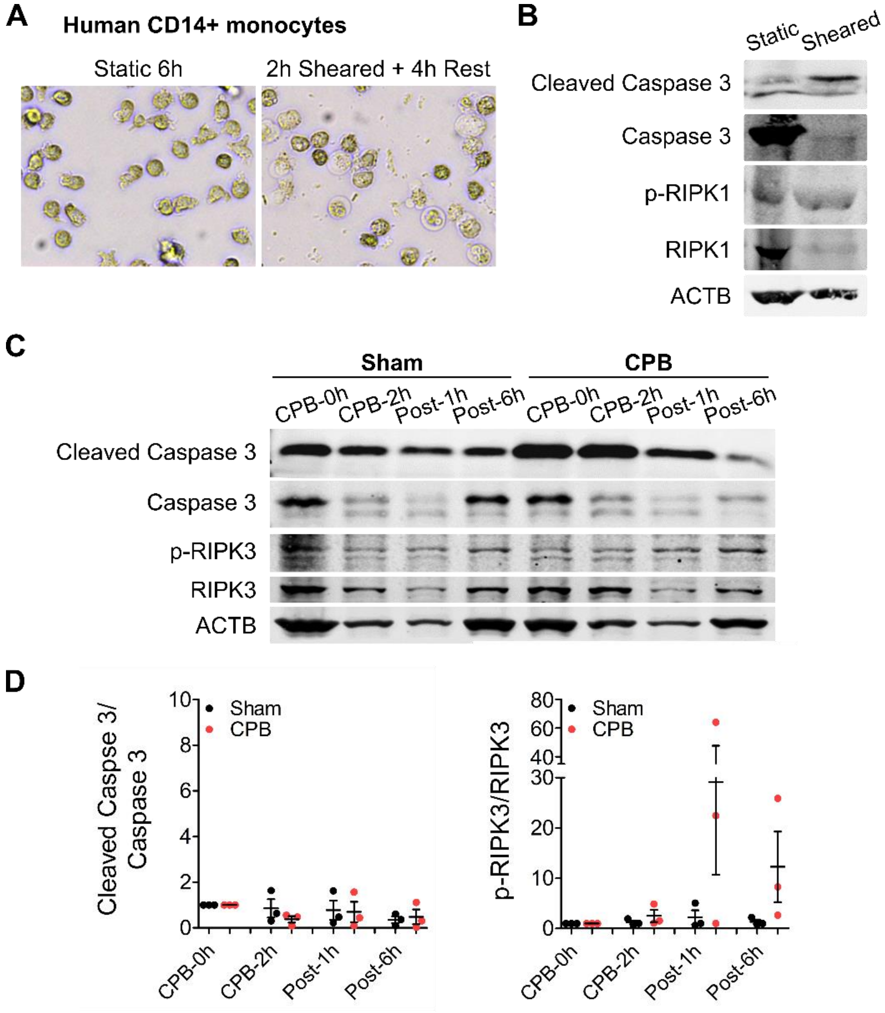

Figure S6. CPB induces cell death in human primary CD14+ monocytes and in leukocytes of bypass piglets. (A) Representative image showed that human primary CD14+ monocytes subjected to the in vitro CPB condition for 2 hrs died within 4 hrs of recovery. **(B)** Western blot showed upregulation of apoptotic marker cleaved caspase 3 and necroptotic marker p-RIPK1 in sheared monocytes compared to static cells. **(C)** Western blot analysis of total leukocytes isolated from sham and bypass piglets at different time points. The level of cleaved caspase 3 seemed to reduce over time in both groups, while the level of p-RIPK3 seemed to increase only in the bypass piglets over time. **(D)** Quantification of western blot images showed no difference in the ratio of cleaved caspase 3/total caspase 3 between the sham and bypass piglets. The ratio of p-RIPK3/total RIPK3 showed a trend of increase at 1 hr and 6 hrs after surgery in the bypass piglets compared to the sham piglets but the values were not statistically significant (n=3 piglets/group). one-way ANOVA and post hoc Tukey's test.

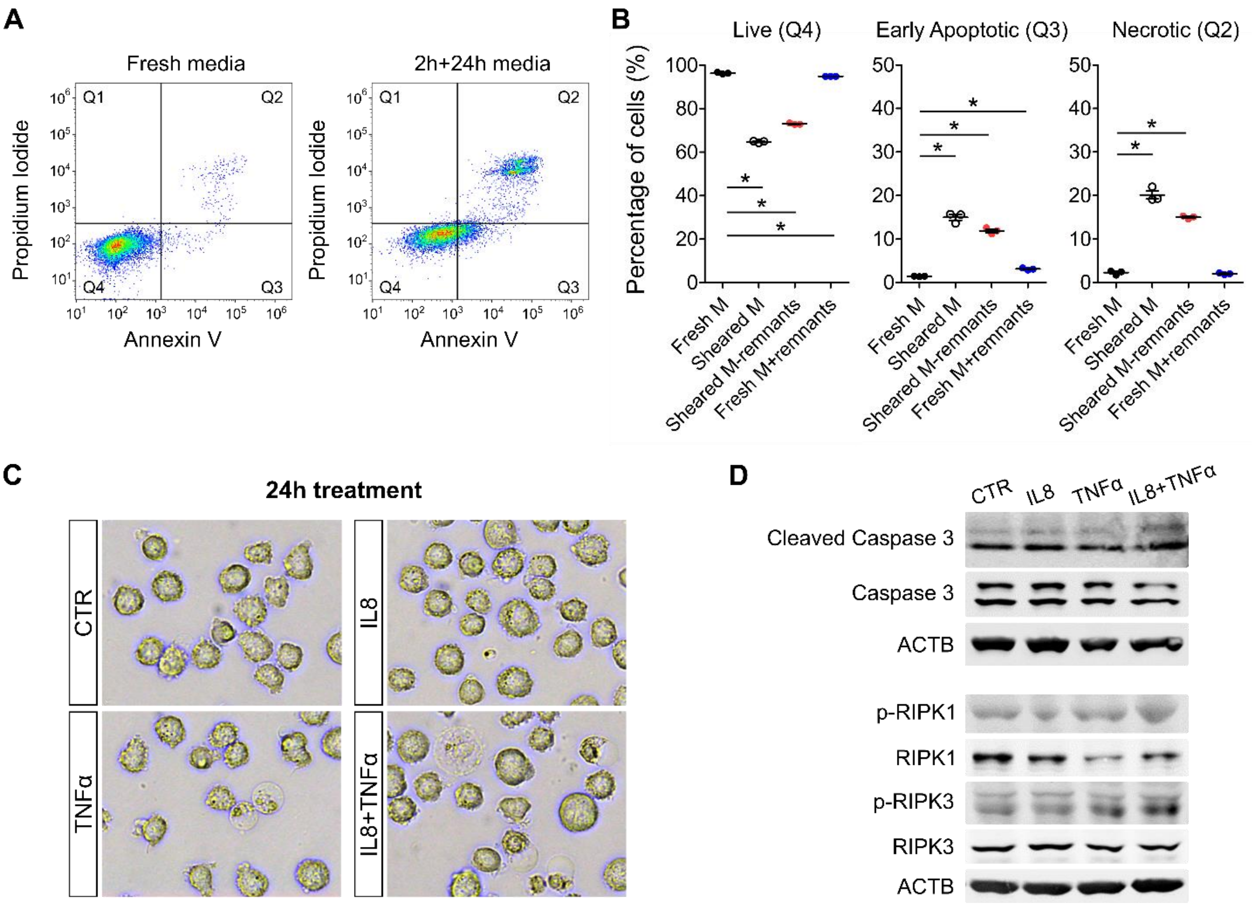

Figure S7. CPB-induced monocytic cell death is mediated by soluble factors in the sheared media. (A) When naïve static THP-1 cells were treated with the sheared media for 24 hrs (media from THP-1 cells sheared for 2 hrs and recovered for 24 hrs), a significant population of cells died as shown in the Q2 quadrant. **(B)** Quantification of the flow cytometry analysis showed significant decrease in the live cell population and increase in the early apoptotic and necrotic cell populations when naïve THP-1 cells were treated with the sheared media for 24 hrs. Removing the remnants from the sheared media or adding the remnants to the fresh media did not affect cell death (n=3 replicates/group). *$p<0.05$, one-way ANOVA and post hoc Dunnett's test. **(C)** Images of naïve static THP-1 cells treated with recombinant human IL8 (200 ng/uL), TNFα (200 ng/uL) or combination for 24 hrs. The combined treatment seemed to cause more cell death than the TNFα treatment only. No cell death from the IL8 treatment was observed. **(D)** Western blot showed that combined treatment of both IL8 and TNFα increased the levels of apoptotic marker: cleaved caspase 3 and necroptotic markers: p-RIPK1 and p-RIPK3 in naïve static THP-1 cells more than the TNFα treatment alone.

Table S1. Patient Demographics

ID	Age (days)	Weight (kg)	Sex	Diagnosis	Lowest temperature (ºC)	Surgery time (min)	Bypass time (min)
**RNA-seq**							
1	7	3.7	M	TGA, ASD, PDA	27.7	277	149
2	14	2.9	M	TAPVC, PDA Secundum ASD	28.2	186	79
3	6	3.0	M	DILV	16.9	253	138
4	19	2.7	F	TAPVC	18.0	163	86
5	12	3.4	F	TAPVC	21.4	173	99
**qPCR**							
1	5	3.3	M	TGA, IVS, ASD	27.4	270	161
2	7	2.7	F	HLHS, PDA, ASD	17.4	320	188
3	4	3.9	M	HLHS, Aortic valve atresia	17.6	295	161
4	23	4.0	M	HLHS, PDA, S/P hybrid	17.6	278	151
5	5	3.2	M	DORV, TGA, IAA+VSD	17.7	344	224
6	33	3.9	M	HLHS	18.0	283	164

TGA – Transposition of the great artery

ASD – Atrial septal defect

PDA – Patent ductus arteriosis

TAPVC – Total anomalous pulmonary venous connection

DILV – Double inlet left ventricle

IVS – Intact ventricular septum

HLHS – Hypoplastic left heart syndrome

S/P hybrid – Status post hybrid

DORV – Double outlet right ventricle

IAA – Interupted aortic arch

VSD – Ventricular septal defect

Table S2. List of SYBR primers

Primers	Species		Sequence
RN18S1	Human	F<br>R	5’ – GGACATCTAAGGGCATCACAG – 3’<br>5’ – GAGACTCTGGCATGCTAACTAG – 3’
IL8	Human	F<br>R	5’ – CTTGGCAGCCTTCCTGATTT – 3’<br>5’ – TTCTTTAGCACTCCTTGGCAAAA – 3’
IL1B	Human	F<br>R	5’ – GAACAAGTCATCCTCATTGCC – 3’<br>5’ – CAGCCAATCTTCATTGCTCAAG – 3’
IL6	Human	F<br>R	5’ – TTCTGTGCCTGCAGCTTC – 3’<br>5’ – GCAGATGAGTACAAAAGTCCTGA – 3’
TNFA	Human	F<br>R	5’ – CCTCTCTCTAATCAGCCCTCTG – 3’<br>5’ – GAGGACCTGGGAGTAGATGAG – 3’
ICAM1	Human	F<br>R	5’ – GTATGAACTGAGCAATGTGCAAG – 3’<br>5’ – GTTCCACCCGTTCTGGAGTC – 3’
SELE	Human	F<br>R	5’ – CAGCAAAGGTACACACACCTG – 3’<br>5’ – CAGACCCACACATTGTTGACTT – 3’
RPL4	Pig	F<br>R	5’ – CAAGAGTAACTACAACCTTC – 3’<br>5’ – GAACTCTACGATGAATCTTC – 3’
IL8	Pig	F<br>R	5’ – TGGACCCCAAGGAAAAGTGG – 3’<br>5’ – TGTTGTTGCTTCTCAGTTCTCT – 3’
IL1B	Pig	F<br>R	5’ – ATGCCAACGTGCAGTCTATG – 3’<br>5’ – TCATGCAGAACACCACTTCTCT – 3’
IL6	Pig	F<br>R	5’ – CAGTCACAGAACGAGTGGATG – 3’<br>5’ – GGACGGCATCAATCTCAGGTG – 3’
TNF	Pig	F<br>R	5’ – TGGCCCAAGGACTCAGATCA – 3’<br>5’ – GGCATTGGCATACCCACTCT – 3’

Table S3. Enriched pathways and biological processes in mRNA-seq DE gene clusters

**Orange Cluster**	**- Log10 (adjusted p)**
Myeloid leukocyte activation	29.03
Response to lipopolysaccharide	6.60
Extracellular structure organization	6.24
Naba_Matrisome associated (genes encoding extracellular matrix)	5.79
Leukocyte migration	5.31
Fra pathway	4.99
Response to toxic substance	4.92
Cytokine production	4.84
Response to purine-containing compound	4.36
IL-17 signaling pathway	4.27
**Yellow Cluster**	**- Log10 (adjusted p)**
Regulated exocytosis	11.25
Post-translational protein phosphorylation	6.55
Cytokine production	6.15
Entry into host cell	5.33
Alcohol biosynthetic process	4.66
Angiogenesis	4.65
Protein processing in endoplasmic reticulum	4.22
Extracellular structure organization	3.60
Leukocyte migration	3.46
Naba_Matrisome associated (genes encoding extracellular matrix)	3.04
**Skyblue Cluster**	**- Log10 (adjusted p)**
Cell cycle	66.58
Cell division	28.14
Cell cycle phase transition	20.39
Mitotic Prometaphase	19.92

Centromere complex assembly	14.25
Factors involved in megakaryocyte development and platelet production	13.66
Aurora B kinase pathway	12.65
G2/M Transition	12.09
Metalloprotease deubiquitinating enzymes	11.52
Regulation of mitotic nuclear division	10.83
**Red Cluster**	**- Log10 (adjusted p)**
Regulation of cytokine production	9.31
Cytokine-mediated signaling pathway	9.29
Cellular response to external stimulus	6.60
Positive regulation of interleukin-6 production	6.42
Myeloid leukocyte activation	6.36
Response to bacterium	6.35
Leukocyte migration	6.27
Regulation of inflammatory response	5.15
Cellular response to mechanical stimulus	5.15
Leukocyte differentiation	5.07
**Blue Cluster**	**- Log10 (adjusted p)**
Immunoregulatory interactions between a Lymphoid and a non-Lymphoid cell	20.02
T cell activation	18.24
IL12 pathway	10.70
Cytokine production	10.01
Adaptive immune response	8.11
Cytokine-cytokine receptor interaction	7.81
Graft-versus-host disease	7.30
Alpha-beta T cell activation	6.50
Regulation of antigen receptor-mediated signaling pathway	5.72
Cellular defense response	4.94

Table S4. Canonical pathways for top cytokines-related biological processes

**Orange Cluster – Myeloid leukocyte activation**	**- Log10 (adjusted p)**
Granulocyte adhesion and diapedesis	7.95
IL6 signaling	5.40
IL8 signaling	5.20
**Yellow Cluster – Cytokine production**	**- Log10 (adjusted p)**
IL10 signaling	8.75
Toll-like receptor signaling	5.08
Acute phase response signaling	4.90
**Red Cluster – Regulation of cytokine production**	**- Log10 (adjusted p)**
Toll-like receptor signaling	9.31
NF-kB signaling	9.29
TREM1 signaling	6.60
